# Shaping inter-brain plasticity: A feasibility study of enhancing inter-brain synchrony with dyadic neurofeedback

**DOI:** 10.1016/j.isci.2026.114894

**Published:** 2026-02-04

**Authors:** Mario Francis, Andrey Markus, Fine Stuhr-Wulff, Simone Shamay-Tsoory

**Affiliations:** 1 Department of Psychology, University of Haifa, Mount Carmel, Haifa 3498838, Israel; 2 The Integrated Brain And Behavior Research Center (IBBRC), University of Haifa, Mount Carmel, Haifa 3498838, Israel

**Keywords:** neuroscience, cognitive neuroscience, psychology

## Abstract

Inter-brain synchrony, the co-activation of brain regions between interacting individuals, is increasingly recognized as a key mechanism underlying social connectedness. This study examined whether dyadic neurofeedback training can enhance inter-brain synchrony in the inferior frontal gyrus and causally increase connectedness. We developed a real-time dyadic neurofeedback platform using functional near-infrared spectroscopy, where unacquainted dyads underwent three training sessions receiving either real neurofeedback or sham feedback. Although we initially anticipated a gradual increase across sessions, the neurofeedback group showed a significant enhancement in IFG inter-brain synchrony only in the third session, with effects extending to additional regions. These neural changes coincided with increased feelings of connectedness and shifts in intra-brain co-activation. While the precise strategies underlying dyadic regulation remain unclear, and additional sessions are needed to fully characterize the learning trajectory, the present findings provide preliminary evidence for the plasticity of inter-brain synchrony and its potential relevance for improving social outcomes.

## Highlights

• A real-time dyadic neurofeedback platform was developed using fNIRS
• Neurofeedback dyads showed increased IFG inter-brain synchrony in the third session
• Neurofeedback-related effects extended beyond the trained brain region
• Higher inter-brain synchrony was accompanied by increased connectedness

## Graphical abstract

**Figure:**
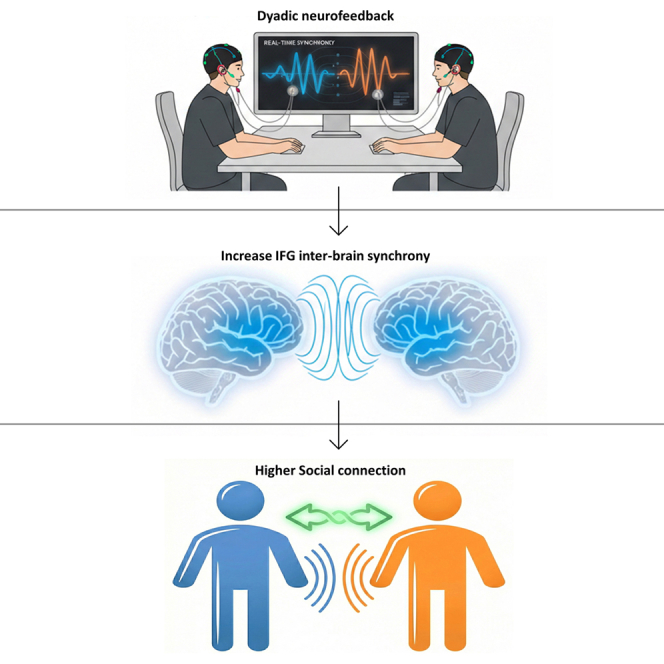

## Introduction

Recently, there has been growing interests in how neural networks of interacting individuals become synchronized during social interactions, forming “extended” networks with inter-brain and intra-brain connections.^1^^,^^2^ The temporal co-activation of brain regions between interacting individuals, known as inter-brain synchrony (or inter-brain coupling), has been identified as a pivotal mechanism supporting social connectedness and facilitating effective interpersonal communication. For example, blood-oxygen- level-dependent signals in the inferior frontal cortex (IFG), measured by functional near-infrared spectroscopy (fNIRS), were found to increase during face-to-face dialog but not during monologue.^3^ Inter-brain synchrony in the IFG was also found to predict movement synchronization,^4^^,^^5^ collaborative decision making^6^ and was associated with performance in a task of interactive song learning.^7^ These findings align with theoretical models identifying the IFG along with inferior parietal lobe (IPL) as critical brain regions within the observation-execution or mirror neuron system,^8^ emphasizing the critical role of these regions in diverse social processes, including imitation^8^ and empathy.^9^ In line with this, a recent meta-analysis identified the IFG, dorsolateral prefrontal cortex (DLPFC), and temporoparietal junction as key regions exhibiting the most substantial effect sizes for inter-brain synchrony during social interactions.^10^

While the observed link between inter-brain synchrony and human connection is compelling, the underlying mechanisms remain insufficiently explored. This gap represents a significant critique in the literature of this burgeoning field. A prominent concern is that neural synchrony might merely be an epiphenomenon arising from individuals performing the same task concurrently.^11^ Although the bidirectional causal relationship between brain plasticity and behavioral changes where each influences the other is well-documented,^12^^,^^13^ it remains unclear whether inter-brain synchrony and human connection are causally linked, and if so, what the directionality of this relationship is.

One method that has been extensively used to explore the causal impact of brain activation on behavior is neurofeedback training. Neurofeedback utilizes the principles of neuroplasticity to generate the reorganization of brain networks based on feedback.^14^ In neurofeedback, participants receive information about their neural activation in a specific brain region associated with a behavioral feature of interest. They are trained to change that activation, allowing for both causal investigation of the relationship between activation and the behavioral feature, and for achieving desired behavioral outcomes.^15^^,^^16^ Neurofeedback is suggested to promote Hebbian-like plasticity,^17^ and to induce long-term neuronal changes.^18^ Theoretical frameworks for neurofeedback hold that operant conditioning and discrimination learning^19^ underly these neuronal changes allowing participants to develop internal strategies to regulate their level of brain activity by selectively reinforcing a specific neuronal change. With respect to behavioral change, neurofeedback involving individual participants was successful in driving behavioral outcomes^20^ ranging from improved attention and working memory,^21^ to stroke rehabilitation,^22^^,^^23^ pain reduction,^24^ and improved emotion regulation.^25^

To date, neurofeedback training has been predominantly applied in individual settings, focusing on the critical objectives of elucidating causal neural mechanisms and modulating behavior. While initial attempts have shown it is possible to connect two participants in one feedback loop,^26^^,^^27^^,^^28^^,^^29^ and create dyadic neurofeedback setting.^30^^,^^31^^,^^32^ Previous dyadic neurofeedback studies have leveraged EEG’s high temporal resolution to demonstrate the potential of neurofeedback to enhance inter-brain synchrony.^33^ These studies employed a variety of innovative feedback stimuli, including light patterns reflecting neural synchrony while setting face to face,^27^ virtual avatars connected by a bridge,^34^^,^^35^ or objects like seesaws and converging balls that responded to shared neural states.^31^^,^^36^ Findings from these studies indicate that dyadic neurofeedback can modulate inter-brain synchrony even within a single session and across different contexts, with some evidence suggesting potential benefits for social outcomes.^34^^,^^35^ Building on this work a critical next step is to localize the neural sources of inter-brain synchrony, as this would clarify which brain systems drive the observed coupling and how these networks relate to specific aspects of social behavior. Such localization requires imaging modalities with higher spatial resolution, even at the expense of temporal precision. However, the causal relationship between inter-brain synchrony and social outcomes remains an open question. Prior studies often lacked pre- and post-intervention behavioral assessments^33^ and frequently included implicit social cues or nonverbal interactions, which may confound interpretations of the observed effects. To draw stronger causal inferences at this stage, future research should employ minimalistic, non-social feedback that isolates the underlying neural mechanisms from social content, along with pre post behavioral designs to assess changes in social outcomes more rigorously.

Prior research suggests that the emergence of inter-brain synchrony is associated with changes in intra-brain synchrony.^37^ As growing evidence suggests that intra and inter-brain synchrony are interconnected,^38^^,^^39^^,^^40^ it is reasonable to speculate that changes in inter-brain synchrony reshape neural pathways within each individual’s brain.^41^ This may indicate that inter-brain synchrony changes specifically in frontal and temporoparietal regions between individuals may change intra-brain connectivity and translate into gains in social interactions.

**Figure 1:**
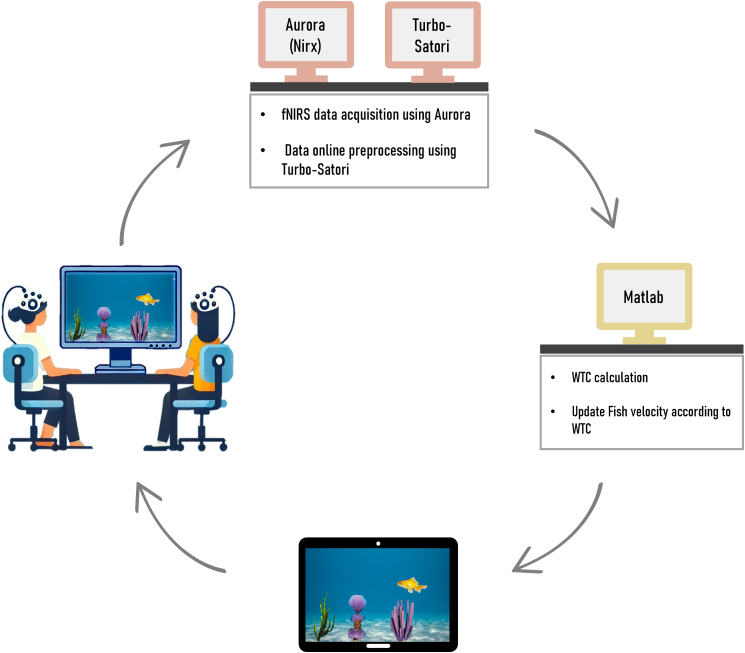
Real-time fNIRS acquisition and dyadic neurofeedback system Real-time fNIRS acquisition and dyadic neurofeedback system: participants sit next to each other facing a computer screen visual stimulation displayed by custom MATLAB software running on a dedicated presentation computer. Wavelet transform coherence (WTC) is calculated based on IFG inter-brain synchrony after being pre-processed using turbo-satori. The feedback includes a swimming fish which changes its velocity to reflect the level of inter-brain synchrony thus serving as neurofeedback.

**Figure 2:**
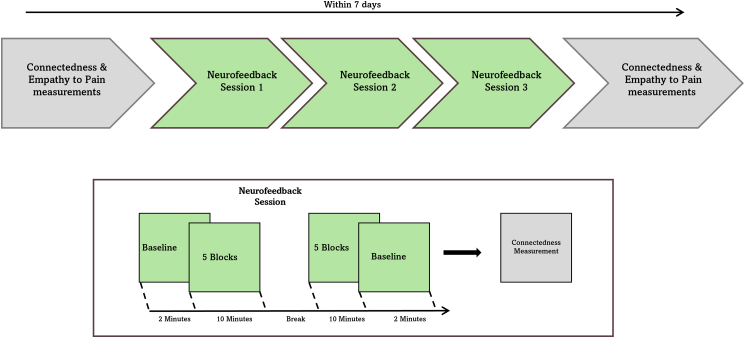
Timeline of the experiment

**Figure 3:**
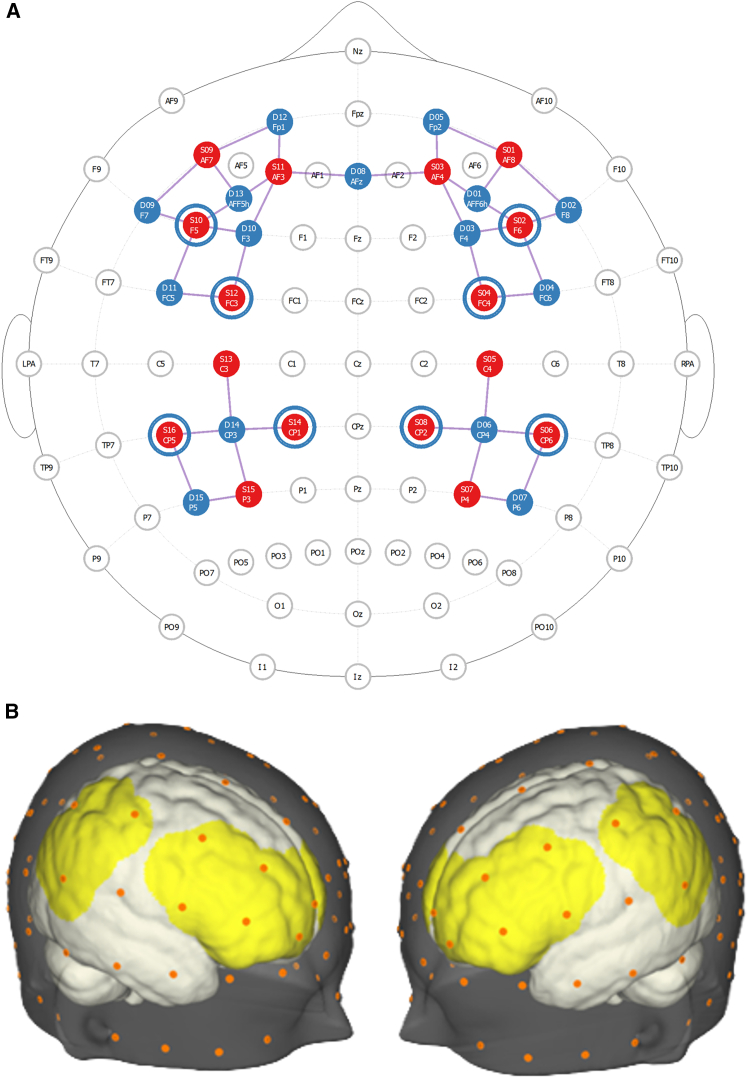
Optode configuration and channel layout The figure illustrates the optode configuration on fNIRS caps (A) Depicts the configuration in 2D using the NirSite application (NIRx), where red circles represent sources, blue circles indicate detectors, and the channels between them are shown. The blue circles surrounding some sources denote short-separation channels. (B) Illustrates the configuration visualized in 3D using Aurora (NIRx).

Building on the inter-brain plasticity theory^2^ and the cell assembly hypothesis,^42^ which posits that neurons across different brains may operate under principles analogous to Hebbian learning—we ask whether inter-brain synchrony can be causally modulated through neurofeedback and whether such modulation yields effects on social connectedness and intra-brain synchrony. We implement a repeated dyadic neurofeedback protocol across three sessions within a one-week interval (For the experimental procedure, please see Figures 1 and 2), targeting inter-brain synchrony in the inferior frontal gyrus (IFG) (however, additional brain regions were also measured during the experiment, see Figure 3), during which the partners were seated in a V-formation facing a shared screen displaying an animated swimming fish. Participants were informed that the fish’s speed was influenced by the combined activity of both their brains and were instructed to increase its velocity as much as possible without speaking or using any form of body language. To preserve natural learning, no specific mental strategies were provided, and participants were informed about the expected delay in feedback due to the hemodynamic nature of the fNIRS signal. Feedback was based either on real-time inter-brain coherence (neurofeedback [NFB] group) or on time-matched prerecorded coherence from another dyad (control group), with participants blinded to their group assignment. Each session began and ended with a baseline period during which the fish remained static, providing pre- and post-session resting measurements for both groups. Perceived interpersonal connectedness was assessed before the first session and after each session (four measurements).

We hypothesized that inter-brain synchrony in the IFG would be gradually upregulated in participants trained to enhance neural synchrony in this region compared to a sham-feedback control group (hypothesis 1). We further predicted that dyads trained to enhance their inter-brain synchrony would report feeling more socially connected and closer to each other (hypothesis 2). Lastly, we explored whether heightened inter-brain synchrony in the IFG would spread into additional regions and would be associated with changes in individual intra-brain connectivity between frontoparietal regions (hypothesis 3).

## Results

### Connectedness scales

To ensure there were no baseline differences in perceived connectedness between groups, a one-way ANOVA was conducted comparing the neurofeedback and control groups at the first measurement. The analysis revealed no significant difference in baseline connectedness between the groups (*F*_(1, 86)_ = 0.22, *p* = 0.64).

**Figure 4:**
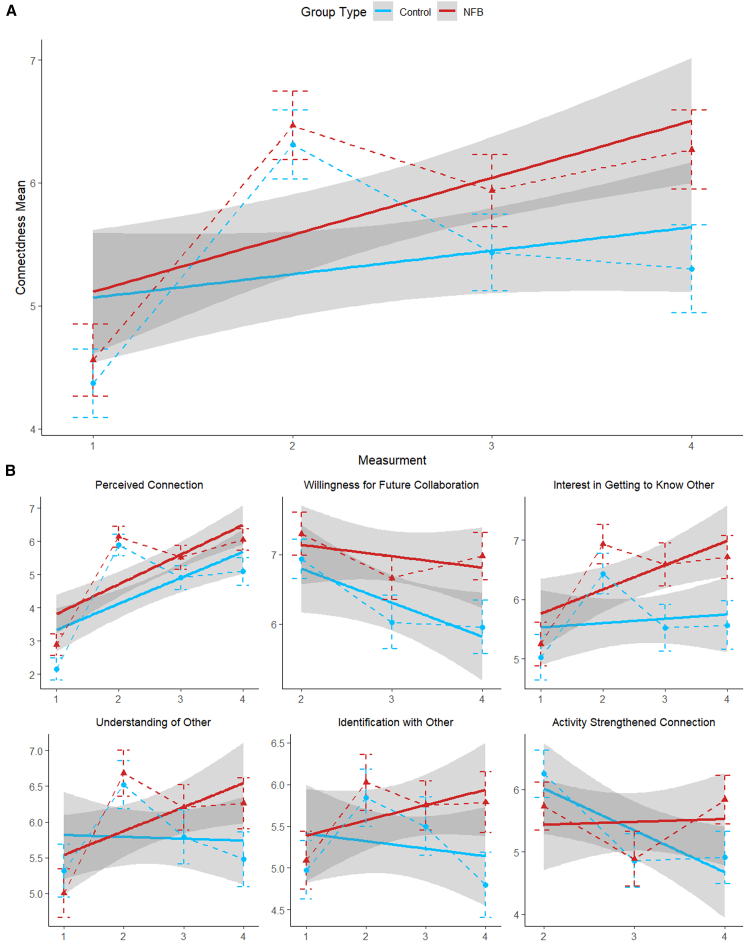
Trends in social connectedness across time by group Trends in social connectedness across time are shown for both the experimental and control groups, with measurement number displayed on the *x* axis (A) presents the overall mean across all questionnaire items, while (B) illustrates the trajectories for each individual item (note that two items were not included in the first measurement). Data were analyzed using linear mixed-effects models with group and measurement as fixed effects and random intercepts for participants nested within dyads. The analysis revealed a significant interaction (F_(1, 258.38)_ = 26.51, *p* = 0.001, η^2^ = 0.09). Error bars represent SD. *n* = 44 dyads (88 participants).

To examine changes in social connectedness over time, we conducted a mixed-effects model analysis with fixed effects of group (experimental vs. control) and measurement (four measurements in total: pre-training and post-training in sessions 1, 2, and 3); random intercepts were included for participants nested within dyads to account for repeated measures; the predicted factor was each participant’s mean score of the items in the connectedness questionnaire (connectedness mean) taken at each measurement. A type II Wald χ^2^ comparison test between the model with interaction terms and the model without interactions revealed that the inclusion of interactions significantly improved the model’s predictive power (χ^2^[1] = 4.9; *p* = 0.027). Thus, the model which included interactions was selected for further analyses. Overall, the connectedness mean showed a significant increase in social connectedness across time points (F_(1, 258.38)_ = 26.51, *p* = 0.001, η^2^ = 0.09). This general increase was observed in both groups (Figure 4A), but the interaction between group and measurement was significant (F_(1, 258.38)_ = 4.90, *p* = 0.028, η^2^ = 0.02). Follow-up analyses revealed that while both groups exhibited positive trends, the experimental group demonstrated greater improvement (M = 0.460, SE = 0.089, t_(258)_ = 5.18, *p* = 0.001) compared to the control group (M = 0.183, SE = 0.088, t_(258)_ = 2.09, *p* = 0.038).

Further analysis of individual items revealed different patterns in participants’ responses in each item (Figure 4B). When asked to what extent they felt connected to the other person or for their willingness to participate in another experiment with the same partner, the interaction was not significant for either question (F_(1, 258.84)_ = 0.36, *p* = 0.55, η^2^ = 0.001) and (F_(1, 170.50)_ = 3.20, *p* = 0.075, η^2^ = 0.02), respectively. For the question assessing participants’ desire to get to know the other person better, a significant group by measurement interaction was detected (F_(1, 258.20)_ = 5.49, *p* = 0.020, η^2^ = 0.02). This interaction reflected a marked positive trend in the experimental group (M = 0.394, SE = 0.097, t_(258)_ = 4.06, *p* = 0.001), whereas the control group showed no significant change (M = 0.074, SE = 0.096, t_(258)_ = 0.78, *p* = 0.44) over time. Similarly, for the question assessing the extent to which participants felt they could understand the other person, a significant group by measurement interaction was observed (F_(1, 258.39)_ = 5.85, *p* = 0.016, η^2^ = 0.02). This interaction was driven by a significant increase in the experimental group (M = 0.338, SE = 0.111, t_(259)_ = 3.05, *p* = 0.003), while no change was evident in the control group (M = −0.039, SE = 0.110, t_(258)_ = −0.36, *p* = 0.72). For feelings of identifying with the other person, the group by measurement interaction also reached significance (F_(1, 258)_ = 4.69, *p* = 0.031, η^2^ = 0.02). This interaction reflected a marginally positive trend in the experimental group (M = 0.180, SE = 0.093, t_(258)_ = 1.95, *p* = 0.052), compared to no significant change in the control group (M = −0.101, SE = 0.091, t(258) = −1.11, *p* = 0.27). Finally, for the question assessing the extent to which the activity strengthened participants’ connection with the other person, the group by measurement interaction was significant (F_(1, 169.33)_ = 7.49, *p* = 0.007, η^2^ = 0.04). This interaction reflected a significant decrease over time in the control group (M = −0.671, SE = 0.186, t_(170)_ = −3.60, *p* = 0.001), while no significant trend was observed in the experimental group (M = 0.057, SE = 0.189, t_(171)_ = 0.30, *p* = 0.77).

### Changes in inter-brain synchrony (neurofeedback analysis)

To verify that there were no baseline differences in inter-brain synchrony between groups prior to neurofeedback training, a one-way ANOVA was conducted on the first baseline measurement. The analysis revealed no significant difference in inter-brain synchrony between the groups at baseline (*F*_(1, 428)_ = 3.18, *p* = 0.08).

**Figure 5:**
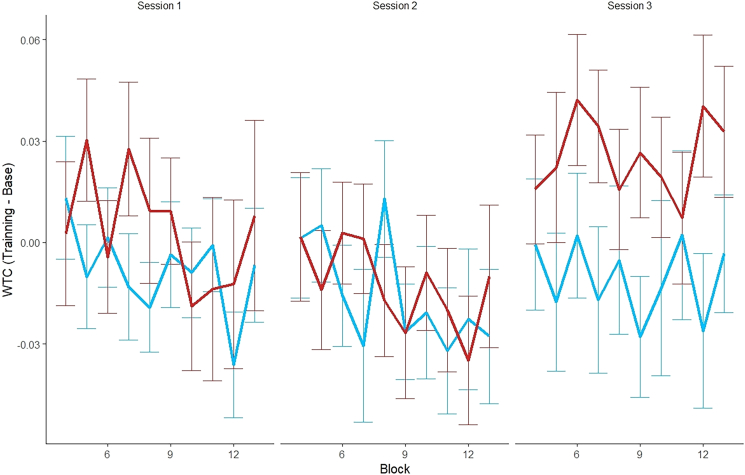
Mean ΔWTC across neurofeedback blocks by session and group Presents the mean ΔWTC for each 2-min NFB block (10 total each session) across sessions and groups the NFB blocks are numbered from 4 to 13, starting after the initial example block and two baseline blocks. Data were analyzed using linear mixed-effects models with group and session as fixed effects and dyad as a random intercept. The analysis revealed a significant interaction (F_(2, 1192)_ = 5.57, *p* = 0.004, η^2^ = 0.01). Error bars represent SD. n = 44 dyads.

To examine changes in inter-brain synchrony between groups in the neurofeedback-trained region (the mean of left and right IFG), a mixed-effects model was employed. We subtracted the mean wavelet transform coherence (WTC) taken in the pre-training baseline of each session from the WTC means for each training block in that session (ΔWTC), in the left and right IFG regions of interest (ROIs). We analyzed changes in ΔWTC across the three sessions for each group. Block number was included as a fixed factor not included in the interactions, to control for variance, and random intercepts were incorporated for each dyad to account for repeated measures. A type II Wald χ^2^ comparison test between the model with interaction terms and the model without interactions revealed that the inclusion of interactions significantly improved the model’s predictive power (χ^2^_(1)_ = 5.52, *p* = 0.019). Thus, the model with interactions was selected for further analyses. The analysis revealed a significant interaction between group and session (F_(1, 1195.37)_ = 5.52, *p* = 0.019, η^2^ = 0.0046), indicating different trends in WTC across sessions for the experimental and control groups. Follow-up analyses examined trends within each group. For the experimental group, the ΔWTC significantly increased across sessions, with a positive trend (M = 0.01026, SE = 0.0037, t_(1196)_ = 2.77, *p* = 0.006). In contrast, for the control group, no significant change in ΔWTC was observed (M = −0.0019, SE = 0.00359, t_(1194)_ = −0.52, *p* = 0.61). To further investigate the source of the significant trend, the session variable was analyzed as a categorical factor using the same linear mixed-effects model. The results revealed a significant interaction between group and session (F_(2, 1192)_ = 5.57, *p* = 0.004, η^2^ = 0.01), thus post hoc analyses were conducted (Figure 5). In session 1, there was no significant difference (M = −0.015, SE = 0.0140, *t*_(63.6)_ = −1.089, *p* = 0.2804) between the control group (*M* = −0.0084, *SD* = 0.0098) and the NFB group (*M* = 0.007, *SD* = 0.01). Similarly, in session 2, the control group (*M* = −0.015, *SD* = 0.0098) and the NFB group (*M* = −0.011, *SD* = 0.0098) showed no significant difference (M = −0.00489, SE = 0.0139, *t*_(61.9)_ = −0.352, *p* = 0.7257). In contrast, session 3 revealed a significant difference between the control group (*M* = −0.012, *SD* = 0.0100) and the NFB group (M = −0.03787, SE = 0.0139, *t*_(61.4)_ = −2.734, *p* = 0.0082) with the NFB group demonstrating a notably higher mean of ΔWTC. To examine differences in inter-brain synchrony across baseline blocks (pre of first session vs. post of third session) and between groups, as well as their interaction, the analysis revealed no significant main effect of group (*F*_(1, 40.83)_ = 0.11, *p* = 0.746), and no significant main effect of block (pre of first session vs. post of third session) (*F*_(1, 39.92)_ = 1.25, *p* = 0.270). Additionally, their interaction was not significant (*F*_(1, 39.92)_ = 1.08, *p* = 0.304).

To evaluate whether the session-3 effect reflects chance rather than learning we contrasted session 3 against the mean of sessions 1 and 2 in the NFB group and extracted the coefficient of this contrast. This contrast (weights: −0.5, −0.5, +1) was chosen because it directly captures the observed pattern in the NFB data, where session 3 consistently exceeded the earlier sessions. The contrast was estimated using a linear mixed-effects model in which the contrast weights served as the predictor and ΔWTC (neurofeedback-related synchrony) was the dependent variable. The model structure matched our primary LME analyses, with block included as a covariate to control for within-session variance and dyad entered as a random intercept.

To test whether this effect could occur by chance, we generated a null distribution by permuting session labels (i.e., session order) within control dyads, while preserving block structure for each session, for 5,000 iterations. For each permutation, the same model was refit and the contrast coefficient extracted, resulting in a null distribution of coefficients. The observed NFB contrast fell in the extreme right tail of this distribution (*p* = 0.0314), providing >95% confidence that the session 3 effect is not a random occurrence but instead reflects a systematic, emergent pattern specific to the NFB group.

### Changes in inter-brain synchrony (offline analysis)

**Figure 6:**
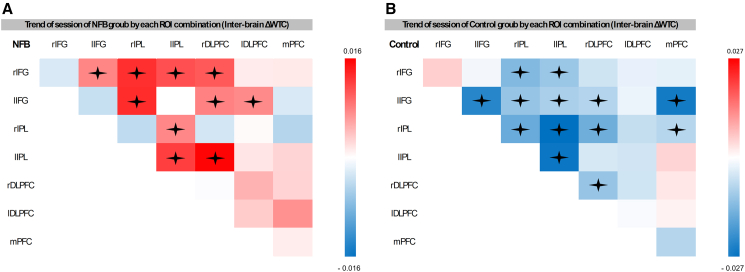
Trend of inter-brain ΔWTC in each ROI combination Illustrates the trend of ΔWTC for each ROI combination in both groups the NFB group (A) and control group (B), represented using a color gradient Red indicates a positive trend, while blue represents a negative trend. Trends close to zero are depicted in white. Statistical trends were estimated using emtrends of linear mixed-effects models including group, session, and ROI combination as fixed effects and dyad as a random intercept. The mixed-effects model revealed a significant three-way interaction between session, group, and ROI (*F*_(27, 56537)_ = 3.44, *p* = 0.0001, η^2^ = 0.0016). Significant trends are marked with an asterisk (*p* < 0.05, corrected). *n* = 44 dyads.

To further examine the effects of IFG inter-brain synchrony neurofeedback training on inter-brain synchrony in other brain regions, the fNIRS signal was divided into the following ROIs: right IFG (rIFG), left IFG (lIFG), right IPL, left IPL, right DLPFC, left DLPFC, and medial prefrontal cortex. Each ROI of participant A was combined with each ROI of participant B within the same dyad, resulting in 7 × 7 = 49 combinations. Since these were not directional, identical ROI pairings, such as lIFG-rIFG and rIFG-lIFG were combined, resulting in a total of 28 combinations. An LME model was constructed with the fixed effects of group (experimental vs. control), session (1, 2, and 3), and ROI combinations (28 in total). Block was included as a fixed variable to control for variance but not included in the interactions. Random intercepts for dyads were incorporated to account for repeated measures. The predicted variable was the ΔWTC. A type II Wald χ^2^ comparison test between a model with all 2- and 3-way interactions, and a model without interactions revealed that the inclusion of the interactions significantly improved the model’s predictive power (χ^2^_(82)_ = 487.26, *p* = 0.001). Thus, the model with interactions was selected for further analyses. The analysis revealed a significant three-way interaction between session, group and ROI (*F*_(27, 56537)_ = 3.44, *p* = 0.0001, η^2^ = 0.0016). Post hoc tests revealed several notable trends in the different ROI combinations (see Table S1 and Figure 6 for a list of ROI combinations in which significant trends were found).

### Changes in intra-brain synchrony

**Figure 7:**
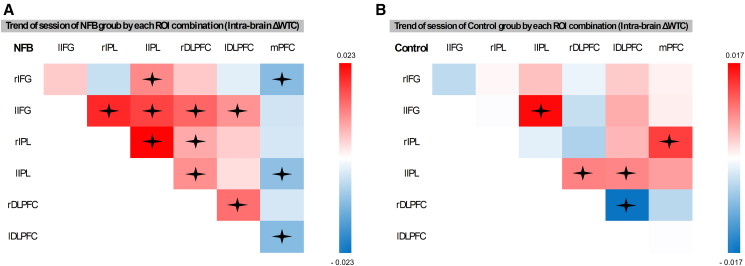
Trend of intra-brain ΔWTC in each ROI combination Illustration of the trend of ΔWTC for each ROI combination in the NFB group (A) and control group (B), represented using a color gradient red indicates a positive trend, while blue represents a negative trend. Trends close to zero are depicted in white. Statistical trends were estimated using emtrends of linear mixed-effects models including group, session, and ROI combination as fixed effects and dyad as a random intercept. The mixed-effects model revealed a significant three-way interaction between session, group, and ROI (F_(20, 97284)_ = 7.8, *p* = 0.001, η^2^ = 0.0016). Significant trends are marked with an asterisk (*p* < 0.05, corrected). *n* = 44 dyads.

To examine changes in intra-brain synchrony (ΔWTC), ROI combinations taken from each participant’s fNIRS recordings were constructed. These combinations excluded homologous ROIs (e.g., lIFG-lIFG), since they have no meaning for intra-brain analyses. Similar to the inter-brain analysis, directionality was not assumed, thus identical combinations, such as lIFG-rIFG and rIFG-lIFG were combined, resulting in a total of 21 intra-brain ROI combinations. LME models were constructed, which incorporated the fixed effects for group (experimental vs. control), session (1 through 3), and ROI combination (21 in total). To account for variance across blocks, block was included as a fixed factor not included in the interactions, and random intercepts for dyads were incorporated to address repeated measures. A type II Wald χ^2^ comparison test between a model with up to a three-way interaction terms and a model without interactions revealed that the inclusion of interactions significantly improved the model’s predictive power (χ^2^_(61)_ = 454.3, *p* = 0.001). Thus, the model with interactions was selected for further analyses. The analysis revealed a significant three-way interaction between session, group, and ROI combinations (ROI combination) (F_(20, 97284)_ = 7.8, *p* = 0.001, η^2^ = 0.0016). Post hoc analyses identified significant ROI combination trends (see Table S2 and Figure 7 for further detail).

### The relationship between inter/intra-brain synchrony and connectedness

The relationship between behavioral change and inter/intra-brain synchrony was examined only in the experimental group, as our primary interest lies in the changes in inter/intra-brain synchrony following the neurofeedback and their association with behavior.

**Figure 8:**
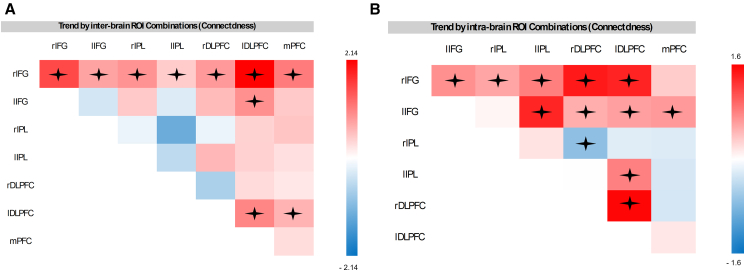
Trends in connectedness as predicted by ΔWTC across ROI combinations Trends in connectedness as predicted by inter-brain (A) and intra-brain (B) ΔWTC across ROI combinations in the experimental group color gradients represent the direction and strength of the trends, with red indicating positive, blue indicating negative, and white indicating trends near zero. Data were analyzed using emtrends of linear mixed-effects models predicting Δconn from ΔWTC and ROI combination, with dyad as a random intercept. Both inter-brain and intra-brain mixed effects model showed significant interaction (F_(27, 27634)_ = 3.4, *p* = 0.001, η^2^ = 0.00332) (*F*_(20, 47570)_ = 4.6595, *p* = 0.001, η^2^ = 0.0045). Significant associations are marked with an asterisk (*p* < 0.05). n = 44 dyads.

To assess the relationship between behavioral change and inter/intra-brain synchrony we created a new variable that represents the change in connectedness. As connectedness was measured four times, we created a new variable by subtracting the mean connectedness score for measurements in the preceding measurement from that of measurements 2–4, resulting in three delta connectedness values (Δconn) representing the change in connectedness between each two consecutive sessions (in other words we had three values of Δconn: [1] connectedness measurement 2 minus 1 [2] 3 minus 2 [3] 4 minus 3). For inter-brain analysis, LME model was constructed to investigate whether changes in the delta of WTC predicted changes in social connectedness following training. The model included Δconn as the predicted variable; ΔWTC, session (corresponding to Δconn measurement), and ROI combination, as fixed factors; the random effects accounted for the intercepts of dyads. While the primary focus was on the prediction of ΔWTC by ROI combinations regardless of session, session was included as a fixed effect to account for variance due to the experimental structure but was excluded from the interactions. A type II Wald χ^2^ comparison test between the model with interaction terms and the model without interactions revealed that the inclusion of interactions significantly improved the model’s predictive power (χ^2^_(82)_ = 318.8, *p* = 0.001). Thus, the model with interactions was selected for further analyses. A significant interaction between ROI combination and ΔWTC was found (F_(27, 27634)_ = 3.4, *p* = 0.001, η^2^ = 0.00332), indicating that the combined effects of WTC changes, and ROI combination significantly predicted changes in social connectedness. Post hoc analysis identified specific ROI combinations showing significant trends (see Table S3 and Figure 8A for the significant ROI combinations).

For intra-brain analysis, an LME model was constructed to investigate whether changes in WTC predicted changes in social connectedness following training. The model included Δconn as the predicted variable; ΔWTC, session (corresponding to Δconn measurement), and ROI combination, as fixed factors; the random effects accounted for the intercepts of dyads. While the primary focus was on the prediction of ΔWTC by ROI combinations regardless of session, session was included as a fixed effect to account for variance due to the experimental structure but was excluded from the interactions. A type II Wald χ^2^ comparison test between the model with interaction terms and the model without interactions revealed that the inclusion of interactions significantly improved the model’s predictive power (χ^2^_(61)_ = 406.2, *p* = 0.001). Thus, the model with interactions was selected for further analyses. A significant interaction between ROI combination and ΔWTC was found (*F*_(20, 47570)_ = 4.6595, *p* = 0.001, η^2^ = 0.0045), indicating that the variation in ΔWTC prediction of changes in Δconn differed across the various ROI combinations. Post hoc analysis identified specific ROI combinations showing significant trends (see Table S4 and Figure 8B for the significant ROI combinations).

### Participants subjective experience

To assess participants’ subjective experiences in each session, we asked them three questions: (1) How much did you enjoy the activity? (2) How much were you able to control the fish at the beginning of the task? (3) And, how much were you able to control the fish at the end of the task? To analyze these responses, we constructed three separate linear mixed models, one for each question. Each model included group and session as fixed factors, with dyad included as a random factor. Participants’ reported ability to control the stimulus at the beginning of the task did not differ significantly between groups (F _(1,252.64)_ = 2.16, *p* = 0.143) or across sessions (F _(1,170.87)_ = 0.24, *p* = 0.627). Additionally, there was no significant interaction between group type and session (F _(1,170.87)_ = 0.95, *p* = 0.332). Similarly, participants perceived control at the end of the task was not significantly influenced by group type (F _(1,195.37)_ = 1.20, *p* = 0.275) or session (F _(1,170.84)_ = 0.12, *p* = 0.728). The interaction between group type and session also failed to reach significance (F _(1,170.84)_ = 2.60, *p* = 0.109). In contrast to perceived control, enjoyment of the activity showed a significant main effect of session (F _(1,170.53)_ = 25.54, *p* = 0.001, η^2^ = 0.13). Participants reported significantly lower enjoyment at the end of the task compared to the beginning. This decrease in enjoyment was observed regardless of group type, as no significant effect of group (F _(1,195.53)_ = 0.37, *p* = 0.544) or interaction between group and session (F _(1,170.53)_ = 0.58, *p* = 0.446) was found.

## Discussion

In this study we sought to examine whether dyadic neurofeedback training targeting inter-brain synchrony in the IFG can enhance inter-brain synchrony in this region, strengthen social connectedness between the participants. Neurofeedback, which traditionally focuses on individual participants, was applied here to pairs of individuals who were trained jointly. Through this design, the study aimed to explore causal mechanisms underlying inter-brain synchrony and social connectedness.

The analysis of inter-brain synchrony demonstrated that dyadic neurofeedback significantly enhanced synchrony in the IFG in the experimental group, but not in the control group. Specifically, participants in the experimental group showed an increase in inter-brain synchrony during the third session, whereas no such pattern emerged in the control group. Importantly, this effect did not extend to the baseline periods: the groups did not differ in the pre-training baseline of session 1 or in the post-training baseline of session 3, nor were there changes between pre- and post-baseline within either group. This outcome is expected, given that neurofeedback trains participants to better synchronize. In contrast, the baseline periods provided no incentive to synchronize such as feedback or social interaction thus the effects were not observed in those segments. Moreover, the pre-training baseline findings confirm that the groups were comparable prior to training and that the divergence observed in the third session arose during the neurofeedback intervention rather than reflecting pre-existing differences. To rule out the possibility that the effects observed in the third session reflect chance and not real learning, we conducted a permutation analysis, which supported that the observed pattern was unlikely to have occurred randomly (see supplemental analysis and Figure S1).

While our initial hypothesis predicted a gradual increase in IFG inter-brain synchrony across sessions, the data revealed that training-related changes became evident only in the final session. This delayed emergence suggests that learning may require multiple exposures or consolidation across days. This aligns with the idea that learning to regulate brain activity through fNIRS feedback is a gradual process. This is likely due to the inherent latency of the hemodynamic response, which can delay the perception and interpretation of feedback signals.^43^^,^^44^ Given the cognitive demands involved, participants may require multiple sessions to effectively associate their internal states with the feedback display and to develop successful regulation strategies. Although the neurofeedback signal in our paradigm reflects hemodynamic activity with an inherent delay of several seconds, extensive evidence indicates that such delays do not prevent learning. fMRI-based neurofeedback studies which operate under the same hemodynamic constraints routinely demonstrate reliable self-regulation despite HRF lag.^14^^,^^45^ Moreover, neurofeedback learning is predominantly implicit, relying on reinforcement of neural states rather than deliberate, time-locked strategies. Participants often learn more effectively *without* explicit strategies,^46^ and learning can even occur when individuals are unaware that neurofeedback is being delivered,^47^ underscoring the role of automatic cortico-striatal reinforcement mechanisms.^48^^,^^49^^,^^50^ Importantly, humans are capable of learning to modulate intra-brain coherence or connectivity based neural signals that they cannot consciously feel or predict,^51^^,^^52^^,^^53^^,^^54^ which closely parallels the synchrony-based target in our dyadic paradigm. Together, this literature suggests that participants do not need to track or anticipate instantaneous fluctuations in the fNIRS signal; instead, the dyad can discover, through trial-and-error reinforcement, the interaction patterns or shared attentional states that tend to produce higher synchrony, even when the underlying signal is delayed or opaque.

This pattern suggests that inter-brain plasticity may require extended exposure and repetition across days before measurable changes emerge, rather than showing a gradual increase at each session. Such a delayed effect is consistent with principles established in single-brain neurofeedback protocols, where repeated training is often critical for inducing neural adaptations, but observable improvements sometimes appear only after multiple sessions.^14^ More broadly, experience-dependent neural connectivity underlies different forms of learning.^55^ According to the principle of spike-timing-dependent plasticity (STDP),^56^ synaptic strength increases when two neurons—or entire brain regions—fire in close succession. Recently, the concept of STDP has been expanded to an inter-brain framework of plasticity,^2^^,^^57^^,^^58^ suggesting that repeated co-activation between brains strengthens inter-brain synchrony through Hebbian-like learning.^2^^,^^42^^,^^57^^,^^58^ Importantly, sleep-dependent consolidation may also have played a role, with intervening nights helping to stabilize and refine the gains, such that synchrony only became evident by the final session. Sleep plays a crucial role in strengthening neural connections and integrating newly learned activity patterns^59^^,^^60^ and in this context, the intervening sleep periods may have enhanced retention of inter-brain synchrony gains. Together, these mechanisms may explain why effects became evident only after three sessions.

The delayed emergence of inter-brain synchrony may also be interpreted through the predictive coding framework. Participants may have required multiple sessions to refine their neural models, gradually reducing prediction errors between expected and actual feedback. By the third session, this iterative error-driven adaptation may have reached a threshold, producing a detectable increase in synchrony.^61^ Importantly, this process was likely reinforced by shared intentionality, as both individuals worked toward the common goal of increasing synchrony. Prior research suggests that inter-brain synchrony is sensitive to shared intentionality, with stronger neural coupling observed when individuals coordinate their efforts toward a mutual objective.^62^ The IFG, a key region in intention coding^63^ may have played a central role in integrating these two mechanisms: participants not only adapted their neural activity in response to real-time feedback (predictive coding) but also did so in the context of a shared goal, which further reinforced synchrony, whereas in the control group the predictive coding did not contribute, since they received sham feedback. Finally, by jointly focusing on the swimming fish as a shared external reference, participants not only trained to synchronize their brain activity, but also developed their ability to maintain coordinated attention. This repeated engagement likely enhanced their capacity for shared attention, a fundamental mechanism underlying effective communication and coordinated behavior.^64^ Over time, this process may have contributed to broader improvements in inter-brain synchrony when the neurofeedback was real. However, the mechanisms by which participants successfully modulated delayed dyadic feedback remain uncertain. Because we did not collect self-reports on strategy use, it is unclear whether participants relied on identifiable cognitive approaches or whether regulation occurred primarily through implicit processes. Future studies should directly assess participants’ strategies through structured debriefing, concurrent physiological measures, or real-time verbal reports to better understand how individuals regulate dyadic neural feedback and how such strategies interact with feedback delay and reward-based learning.

A detailed analysis of inter-brain synchrony revealed that the enhancement primarily involved increased inter-connectivity between the IFG and IPL, as well as their synchrony with the DLPFC, compared to the baseline. In contrast, the control group showed no significant upregulation in these regions but instead exhibited a decrease in inter-brain synchrony within the frontoparietal areas. Both the IPL and IFG are critical components of the observation-execution system, a system with robust empirical evidence for its role in a range of social interactions and phenomena.^2^^,^^8^^,^^9^ Thus, it is not surprising that manipulating inter-brain synchrony in the IFG also extended to increased inter-brain synchrony in the IPL and between these regions. This pattern further supports the interpretation that genuine learning emerged by the third session as opposed to chance, given the parallel effects in related regions. Considering the evidence that neurofeedback successfully enhanced inter-brain synchrony, particularly in the IFG, an important question arises: does this enhancement translate into positive social outcomes?

In line with our hypothesis, we found that the experimental group demonstrated a significantly steeper increase in feelings of social connectedness compared to the control group, particularly on measures related to understanding, identification, and a desire to get to know one another better. This supports the view that inter-brain synchrony plays a role in fostering social closeness. Notably, items specifically addressing “feeling connected” or a willingness to re-engage in the experiment also showed positive trends, though their interactions did not consistently reach significance. This may reflect a ceiling effect or a baseline willingness among participants to cooperate in a controlled laboratory setting. Unexpectedly, ratings on items measuring willingness for future collaboration and perceived strengthening of connection decreased in the control group, while remaining stable in the neurofeedback group. One possibility is that the mental load required by the task may have led to fatigue and reducing participants’ motivation to participate in possible subsequent meetings. The participants may have interpreted this question as relating to their overall participation in the experiment. While a significant reduction was observed in the control condition, this decrease was attenuated in the experimental condition, likely due to the general increase in perceived connectedness. Crucially, changes in inter-brain synchrony—specifically the right IFG’s inter-connectivity with frontoparietal regions—explained a significant portion of the variance in the improvement of social connectedness over time. In line with this association, the behavioral measures also showed a stronger group difference at the fourth measurement (third session), reinforcing the view that meaningful learning emerged during the final session. Importantly, these effects cannot be attributed to differences in participants’ subjective experiences of control or success, as no significant group differences were observed on these measures. This finding is consistent with prior literature suggesting that a substantial portion of the neurofeedback learning process occurs implicitly.^14^ Another possible explanation is that, since participants only achieved measurable success by the third session, they may not have yet developed a conscious sense of control.

While prior hyperscanning studies have shown correlational links between inter-brain synchrony and diverse social behaviors,^38^^,^^41^^,^^65^ the field has lacked direct causal evidence demonstrating whether neural synchrony can be reliably enhanced and whether such changes meaningfully impact social outcomes. The results in this study show that increases in inter-brain synchrony were significantly associated with enhanced social connectedness. This association provides preliminary casual insights: a manipulation of dyadic synchrony in frontoparietal regions not only correlates with social closeness but may actively contribute to shaping it. These findings reinforce the idea that inter-brain synchrony supports social connectedness, aligning with its proposed role in the mirror neurons system and social cognition.^8^^,^^66^

A key finding of this study was the association between neurofeedback training targeting inter-brain synchrony in the IFG and significant changes in intra-brain connectivity patterns. In the experimental group, participants showed enhanced intra-brain synchrony between the IFG and IPL, alongside DLPFC and a notable diminished synchrony with mPFC. In contrast, the control group exhibited the opposite pattern: mostly the IFG-IPL connections, except for the left IFG-left IPL connection, were nonsignificant, while intra-brain synchrony between prefrontal areas and the IPL increased compared to baseline. These contrasting patterns could reflect distinct neural adaptations to the feedback conditions. The enhanced IFG-IPL synchrony observed in the experimental group aligns with the established role of these regions in the observation-execution (mirror neuron) system, which supports shared attention and action understanding. This suggests that successful inter-brain synchrony training may have reinforced these pathways to facilitate effective social coordination. Additionally, given the DLPFC’s role in cognitive flexibility,^67^^,^^68^ it may have operated in tandem with the IFG and IPL to develop and adapt synchronization strategies when participants received true feedback.

In contrast, the increased synchrony observed in the mPFC, DLPFC, and IPL within the control group—where dyads failed to synchronize—aligns with the mPFC’s role in the default mode network (DMN), which supports internally directed cognition.^69^ The mPFC has been implicated in processing social misalignment, particularly through increased DMN engagement when individuals struggle to establish synchrony, this suggests heightened introspection and self-generated predictions about a partner’s responses.^70^^,^^71^^,^^72^^,^^73^^,^^74^ Additionally, the DLPFC’s role in flexible cognition,^67^^,^^68^ and the IPL observation role in the observation-execution system,^75^ particularly in sensory-to-action mapping between one’s own body and external coordinates thus activating during both compatible and incompatible coordination,^76^ along with its broader involvement in social phenomena,^9^ suggests that these regions, may have operated in tandem as a compensatory mechanism to manage the lack of successful synchrony, potentially facilitating more internal focused reflection. This heightened synchrony reflects increased reliance on internally focused and effortful cognitive processes in the absence of successful interaction, aligning with the notion that IPL nodes are associated with the DMN^77^ and mind-wandering.^78^

This likely suggests that when inter-brain synchrony was successfully achieved, participants relied less on individual executive function and internally focused processing. Instead, their brains shifted toward more efficient use of inter-brain synchrony in the observation-execution system, including the IPL, to support shared cognitive and attentional states. These findings may indicate that dyadic neurofeedback training has the potential to reshape both interpersonal and intrapersonal neural connectivity and functions. This aligns with prior work suggesting that shifts in inter-brain synchrony are closely linked to corresponding changes within each individual’s brain networks.^37^^,^^38^^,^^39^^,^^40^^,^^79^

Taken together, the findings of the current study provide preliminary evidence indicating that an intervention that is aimed at increasing inter-brain synchrony may simultaneously increase key social capacities such as connectedness through modulations of individual neural pathways. Since frontoparietal synchrony is shown to enhance social connectedness, these findings could have significant applications in therapeutic and educational contexts. For example, group-based or dyad-based neurofeedback might help individuals with social cognitive deficits, such as those with autism spectrum conditions, by strengthening the neural networks that support social interaction. Beyond the current study, inter-brain synchrony has been linked to successful teamwork and collaborative problem-solving.^80^ Dyadic neurofeedback could therefore be adapted to improve team cohesion in work or sports settings by training groups to achieve higher levels of neural synchrony, ultimately fostering more effective interpersonal coordination.

Taken together, the present findings reveal the feasibility and promise of dyadic neurofeedback as a tool for shaping inter-brain synchrony and its associated social outcomes. Demonstrating that repeated neurofeedback training sessions can selectively enhance IFG inter-brain synchrony, our study offers direct causal evidence linking shared neural activity to increased social connectedness. These results pave the way for future research to explore dyadic neurofeedback across diverse interaction contexts (e.g., cooperative vs. competitive tasks), interpersonal dynamics (e.g., groups of three or more), and clinical applications (e.g., social anxiety interventions and social skills development).

### Limitations of the study

Despite these encouraging results, several methodological considerations warrant caution. As a proof-of-concept investigation, this study was designed to establish the feasibility and initial efficacy of our novel neurofeedback protocol. Future studies with larger and more diverse samples are needed to confirm and extend these findings. Moreover, during the neurofeedback sessions, participants did not engage in any specific behavioral tasks other than jointly observing the stimuli. As a result, the higher inter-brain synchrony observed in the experimental group may have arisen spontaneously, rather than from shared strategies developed through the training. Relatedly, because we did not collect self-reports of participant strategies, the mechanisms underlying successful regulation remain unclear. Additionally, while fNIRS offers better spatial accuracy compared to other techniques, it introduces latency in detecting brain activity.^81^ This latency varies between individuals, meaning that the feedback stimuli reflected neural activity that occurred a few seconds earlier in both brains, potentially misaligning the timing of the feedback. Additionally, the current study has low number of male participants, which may limit the generalizability of the findings across genders. Future studies should aim for a more balanced gender distribution to examine potential sex differences in neurofeedback response and inter-brain coupling. Another limitation is that given that the significant change in inter-brain synchrony emerged only in the third session, an additional session may be necessary to more fully capture the learning trajectory. Future studies should consider this in their design. Furthermore, the scope of our follow-up measures was relatively short. While we observed promising changes after three sessions within one week, the durability of these changes beyond the immediate training period is unknown. Longitudinal studies examining retention over weeks or months are needed to determine whether inter-brain plasticity persists and continues to influence social behavior over time. Methodologically, we used a shorter sliding window in our WTC analysis, which is well-suited for detecting faster oscillations that may be associated with implicit learning processes. In contrast, longer windows may better capture slower dynamics linked to explicit learning.^82^ Finally, this study did not examine whether the effects of the training generalized beyond the dyadic context to broader social outcomes. Future research should investigate whether such effects extend to capacities such as empathy.

## Resource availability

### Lead contact

Further information and requests for resources should be directed to and will be fulfilled by the lead contact, Mario Francis (mario.f_97@hotmail.com).

### Materials availability

This study did not generate new materials.

### Data and code availability

• Raw and preprocessed data have been deposited at Mendely Data and are publicly available as of the date of publication at Mendeley Data: https://doi.org/10.17632/wyc43xpcff.1.
• All original code has been deposited Mendely Data and are publicly available as of the date of publication at Mendeley Data: https://doi.org/10.17632/wyc43xpcff.1.
•Any additional information required to reanalyze the data reported in this article is available from the lead contact upon request.

## Acknowledgments

The work was supported by the 10.13039/501100000781European Research Council grant (INTERPLASTIC: 101020091; DLV-101020091).

## Author contributions

M.F.: design of the research, performance of the research, data collection, data analysis, writing the manuscript, and final approval; A.M.: design of the research, data analysis, writing the manuscript, and final approval; F.S.-W.: design of the research and final approval; S.S.-T.: design of the research, writing the manuscript, supervision, and final approval.

## Declaration of interests

The authors declare no competing interests.

## Declaration of generative AI and AI-assisted technologies in the writing process

During manuscript preparation, generative AI tools were used for language refinement and proofreading. The authors critically reviewed and edited all content and take full responsibility for the final version of the manuscript.

## STAR★Methods

### Key resources table

**Table:** 

REAGENT or RESOURCE	SOURCE	IDENTIFIER
**Software and algorithms**		

Neurofeedback Code	Self-developed; this study	https://doi.org/10.17632/wyc43xpcff.1
Turbo-Satori	Brain Innovation, Maastricht, NL	https://www.brainvoyager.com/company/company.html
Satori	Brain Innovation, Maastricht, NL	https://www.brainvoyager.com/company/company.html
Matlab R2023b	MathWorks	https://www.mathworks.com/products/matlab.html
Qualtrics	Qualtrics international	https://www.qualtrics.com/

### Experimental model and study participant details

Ninety-eight Israeli participants (aged 18-35; see Table S7 for detailed demographics of the analyzed participants) were recruited from the University of Haifa student community. Participants were randomly assigned, upon confirmation of all inclusion criteria, to either the dyadic experimental group (25 same-sex dyads; 3 male, 22 female) or the control group (24 same-sex dyads; 5 male, 19 female). The target sample size was 44 dyads (22 per group), based on an *a priori* power analysis assuming small-to-medium effect sizes (η^2^ = 0.04), α = .05, statistical power ≥ .95, and a correlation of .5 between repeated measures. Race and ethnicity were not formally assessed in this study. Instead, participants reported their mother tongue as part of the demographic questionnaire. Inclusion criteria required participants to be over 18 years of age, have no history of neurological or psychiatric disorders, and be willing and able to provide informed consent. Exclusion criteria included a dominant left hand (as left-handed individuals may exhibit greater variability in brain organization^83^), current substance abuse or dependence, significant or unstable medical conditions that could affect study outcomes, and inability to comply with study procedures or provide informed consent. To increase homogeneity within dyads, participants were randomly paired into same-sex dyads, and the level of familiarity was controlled such that participants reporting prior acquaintance were not assigned to the same pair. Three dyads were excluded due to participant dropout by one or both members, and two additional dyads were excluded because one participant consistently arrived late to every session, creating a tense and potentially confounding interaction. As a result, 22 dyads per group were included in the final analyses (see Figure S2 for the CONSORT diagram and Table S7 for demographic details). This study was conducted as part of a larger project on dyadic neurofeedback and complied with all relevant ethical regulations. Ethical approval was obtained from the University of Haifa Ethics Committee (approval number: 0263/21). All participants provided written informed consent and received monetary compensation of 150 NIS for completing the three neurofeedback sessions.

### Method details

#### Experiment procedure

The neurofeedback training involved three sessions conducted over one week, spaced 24–48 hours apart. Each session included 10 blocks, with a 2-minute duration per block, and a short break between blocks 5 and 6. A 2-minute baseline was recorded both before the first block and after the last block. Each training session lasted approximately 24 minutes, with 20 minutes dedicated to active neurofeedback regulation. At the first session, participants were welcomed into a private room where they met their dyad partner for the first time. Following a brief introduction, both participants were seated facing the same direction, with a computer monitor in front of each. They were asked to complete the connectedness questionnaire displayed on the screens. After completing the initial questionnaire, participants were fitted with fNIRS caps, and their seating was adjusted into a V-shape, oriented toward a central screen. The neurofeedback task was then introduced: an animated swimming fish appeared on the screen, and participants were informed that the speed of the fish was influenced by the joint activity of their brains. They were instructed to refrain from communicating with one another during the task and to focus solely on the screen. It was further explained that the brain activity measurements utilized BOLD signals, which introduced a slight delay between changes in brain activity and their reflection on the screen. This delay was emphasized to ensure participants understood that visual feedback was expected to be delayed by a few seconds. Participants were blinded to their group assignment (real vs. sham feedback). A 2-minute baseline was recorded, during which the fish remained stationary, followed by the start of the neurofeedback task, which lasted for 20 minutes, divided into 10 blocks with breaks between each pair of consecutive blocks. One of the participants was instructed to press a key to end each break once both were ready. A longer break was introduced between the 5th and 6th blocks, during which participants were asked to call the experimenter. During the task, participants attempted to increase the speed of the fish by synchronizing their brain activity. After completion of the task, another 2-minute baseline was recorded. The fNIRS caps were then removed, and the computer screens were adjusted to allow each participant to complete the connectedness scales questionnaire individually. The second and third sessions were conducted at least 24 hours apart from the first session and from each other. Each session included a neurofeedback component comparable to the first, followed by completion of the connectedness questionnaire.

#### Neural data acquisition

We used the NIRx fNIRS system to simultaneously measure cortical oxygenated hemoglobin (O_2_Hb) and deoxygenated hemoglobin (HHb) concentrations from dyads. The NIRx system employs 16 light emitting sources and 23 detectors, composing a total of 46 fNIRS channels. From these channels 38 were regular “long separation” channels, characterized by a distance of 30 to 45 mm between the light source receiver. Thus, these channels were able to measure hemoglobin concentration changes, reflecting blood oxygenation levels, at a depth of 15-20 mm. below the surface of the cortex, in addition to extracerebral signals originating from the extraneous tissue.^84^ Eight of the channels were short separation channels, limited in penetration depth to measuring only the extracerebral signals. The latter include blood pressure fluctuations, such as Mayer waves, respiration and cardiac cycles, which affect the blood flow in the cortex and the extracerebral tissue. Therefore, the signal measured by these channels could be used to regress superficial components from the fNIRS signal, allowing isolation of the cortical functional response.^85^ The channels, or optodes, were placed over the medial prefrontal cortex (mPFC), dorsolateral prefrontal cortex (dlPFC), inferior frontal gyrus (IFG), inferior parietal lope (IPL) (See montage in Figure 3). Optode placement follows the international 10–20 system anchor points. Optode coordinates were determined using the Aurora Program by NIRx. To create these optode placements according to the desired brain regions of intrests, the fNIRS Optodes' Location Decider (fOLD)^86^ was used based on the specified ROIs according to Broadman atlas – for the MNI coordinations and landmarks (see Tables S5 and S6). The fNIRS placement accounted for different head sizes by fitting each participant with a suitable cap determined by their head diameter. Continuous wave fNIRS was used to assess cortical hemodynamic activity, utilizing two wavelengths 760 nm and 850 nm sensitive to deoxygenated and oxygenated hemoglobin, respectively. Measurement of oxygenated hemoglobin (O2Hb) concentrations was obtained at a sampling frequency of ∼5 Hz using Aurora software. These raw fNIRS data were used for generation of neurofeedback values in real-time and were also saved for later online analyses.

#### Dyadic neurofeedback setup

During the feedback sessions, imaging data was acquired from both participants in a dyad simultaneously using two fNIRS systems. Online preprocessing was carried out using Turbo-Satori (Brain Innovation, Maastricht, NL). Preprocessed data from both participants were collated, and a coherence measure was continuously calculated throughout the session. This measure was used to modulate a visual feedback display, completing the neurofeedback loop. The visual feedback consisted of an animated swimming fish, the velocity of which varied in response to the coherence measure. Participants were divided into two groups. In the neurofeedback group, the velocity of the fish was directly linked to their real-time coherence values, with higher coherence resulting in greater velocity. In contrast, the control group received sham feedback, where the velocity of the fish was based on time-matched coherence values from a different dyad. This approach ensured the feedback appeared realistic while remaining unrelated to the participants' actual brain activity. In both the experimental and control groups participants were instructed to jointly increase the velocity of the fish, applying the strategy they feel is the most successful. Other than this, no specific instructions regarding mental strategy were given, because neurofeedback studies showed that an explicit strategy is not required^19^ and may even impede learning to regulate brain activation.^87^ In both groups, real coherence values were calculated and retained for further analyses.

#### Visual feedback

The visual portion of the feedback loop was comprised of a short clip of a fish facing leftwards and moving slightly around the center of the screen, placed against a moving background of sand, rocks and marine flora. This generated an illusion of a fish swimming in the ocean from the right to the left of the display. The speed at which the background moved across was determined by the received neurofeedback values. Thus, the overall effect was that of a fish swimming faster when coherence values were higher (either real or sham, depending on the group). Both participants in a dyad were seated in front of a single computer monitor presenting this visualization, thus receiving identical concurrent feedback.

### Quantification and statistical analysis

#### Online preprocessing

fNIRS data collected by each of the two NIRx devices during the neurofeedback sessions were streamed for preprocessing to the Turbo-Satori platform, with the pre-processed signal becoming available in near real-time. Pre-processing included online frequency filtering using a bandpass filter with a 0.2 Hz. low cutoff and a 0.01 Hz. high cutoff frequencies, and short-separation regression on all available fNIRS channels. Preprocessing aimed at removing extracerebral physiological signal artifacts, such as those resulting from heartbeat and respiration. Additionally, Turbo-Satori converted raw light intensity values collected by the NIRx devices into relative O_2_Hb and HHb concentrations using the modified Beer–Lambert law.^88^^,^^89^ Subsequent to this, a preprocessed data stream was generated, which was used in neurofeedback signal computation. This online preprocessing was applied only to the IFG signal, the target of neurofeedback, as extending it to all measured regions would delay the feedback, which is undesirable for the learning process. Therefore, offline preprocessing was performed later for the other regions.

#### Coherence computation

Custom Matlab (Matlab v.2023b, by Mathworks Ltd.) code was used to combine the preprocessed data streams from both participants in order to calculate coherence values. The code was designed to run in near-real time. First the code read new timestamped data samples from each data stream as new data frames arrived. Then, only data for the oxygenated hemoglobin (O_2_Hb) concentrations from the relevant regions of interest (ROIs) was retained from each frame. The ROIs chosen for neurofeedback computation included the left and right Inferior Frontal Gyri (IFG). Data samples related to the IFG of each participant were averaged within each new frame. Samples from the two streams were combined by timestamp into a joint structure, which represented a synchronized dyadic dataset of the IFG of each participant. Coherence values were calculated from this set using Wavelet Transform Coherence (WTC) with a Morlet seed wavelet^79^ with wavelengths of between 6 and 30 seconds. The wavelength for WTC analyses were determined from analysis of the control group in a pilot study and were constrained by the length of the computation window. The WTC toolbox^90^ for MATLAB was used for this calculation. The calculation was performed at 1 seconds. intervals asynchronously to the arriving data, and a backward window of 30 seconds from the most current dataframe’s timestamp was used for each calculation cycle. Since data frames arrived at a frequency of ∼5 samples-per-second, each new calculation cycle included between 4-6 new data samples, omitting the same number of the oldest samples. Coherence values resulting from each computation cycle were averaged across time and wavelength to produce a single neurofeedback value, which was transmitted to the feedback visualizer for the neurofeedback group. Additionally, the code had the ability to load a data file pertaining to a previous session of a different dyad and transmit coherence values from that file to the feedback visualizer for the control group. Same as the preprocessing the online WTC calculations was applied only to the IFG signal, the target of neurofeedback, as extending it to all measured regions would delay the feedback, which is undesirable for the learning process.

#### Offline analyses

In addition to the calculated coherence values, raw data from each neurofeedback session were retained for offline analyses, regardless of the group to which each dyad was assigned. This was done in order to allow for a more fine-grained analysis of brain activation during the sessions, including ROIs which were not included in the coherence calculation, as well as separating the components of the left and right IFG and other brain areas. Additionally, this allowed us to examine longer wavelet lengths than those used in the online computations.

#### Offline data preprocessing

Offline data preprocessing was performed using Satori (Brain Innovation). Raw intensity data were first converted to optical density values. Motion artifacts were corrected using TDDR and spike removal procedures. To minimize physiological noise while preserving hemodynamic signals, a bandpass filter (0.01–0.2 Hz) was applied. Signal quality was assessed with the Scalp Coupling Index (SCI), which quantifies the presence of cardiac pulsations by calculating the correlation between oxy- and deoxy-hemoglobin signals in the heartbeat frequency band. Channels with SCI values below 0.75 were excluded from further analysis. For the remaining channels, short-separation regression was performed using the nearest short-separation channel as a regressor. The filtered optical density data were then transformed into concentration changes of O_2_Hb and HHb using the modified Beer–Lambert law.

#### Wavelet transform coherence (WTC)

We used WTC to assess inter and intra-brain synchrony offline similarly to the online computation, but with slightly different parameters. WTC analyses focused on frequencies between 0.015 Hz (period = 66 seconds) and 0.16 Hz (period = 6 seconds) to target the hemodynamic response excluding noise from breathing or cardiac activity. We calculated coherence for each pair of homologous ROIs (e.g., left IFG of Participant A and left IFG of Participant B) across dyads, and the coherence between all possible ROIs in individual brains, using MATLAB's Wavelet Coherence Toolbox.

#### Social connectedness assessment

##### State connectedness scales

To assess the level of state connectedness between participants we developed a state connectedness scale based on the social connectedness questionnaire,^91^ which measures several aspects of connectedness including bonding, understanding, and approach. Three items assess bonding (e.g., “To what extent do you feel connected to the other person?”); two items assess approach motivation (e.g., “To what extent would you like to participate in another experiment with the other person?”), and one item assessing understanding (e.g., “To what extent do you feel you can understand the other person?”). The items were presented in a questionnaire using the Qualtrics (Qualtrics international, Inc.) platform. Each item was measured on a 0 to 10 scale. A score representing overall connectedness was calculated by averaging the responses to all the items.

#### Subjective experience of control

After each session, participants rated how successfully they were able to control the speed of the fish at both the beginning and the end of the session. Ratings were provided on a scale from 0 (not at all) to 10 (completely successful).

#### Statistical analysis

Analyses were performed using linear mixed-effects (LME) models to account for the nested structure of the data and repeated measures design.^92^^,^^93^ Model fitting was conducted using the maximum likelihood estimation method utilizing the lme4 package in R software environment.^94^ Post hoc analyses, using the EMMEANS package for R,^95^ were conducted to explore significant effects and interactions, and effect sizes were reported where relevant. All tests controlled for multiple comparisons using Tukey's method where applicable. For the behavioral analysis we examined changes in social connectedness using LME with fixed effects for group (experimental vs. control), session, and the interaction between them. Random intercepts for participants nested within dyads were included to account for repeated measures. To examine changes in levels of connectedness, new variables representing session-to-session changes were created and included in the models to investigate associations with neural measures.

For the neuroimaging data we analyzed changes in inter-brain synchrony (subtracting the mean WTC taken in the pre-training baseline of each session from the WTC means for each training block in that session [ΔWTC] in the left and right IFG ROIs) across sessions and ROIs, using mixed-effects models. Fixed effects included group, session, ROIs, and their interactions. Random intercepts for dyads were included to control for repeated measures. Separate models were developed to analyze (1) inter-brain synchrony between all ROI combinations and (2) intra-brain synchrony within individuals, incorporating all ROI combinations.

To assess brain-behavior relationships we constructed additional LME models, which examined the relationship between changes in inter- and intra-brain synchrony (ΔWTC) and in social connectedness in the experimental group. These models included ΔWTC and ROI combinations, and their interactions as fixed effects, with random intercepts for dyads.

Model selection was performed using Type II Wald χ^2^ likelihood-ratio tests, comparing models with and without interaction terms. When interactions were significant, post hoc analyses and trend estimates were conducted. Degrees of freedom were estimated using Satterthwaite’s approximation. Effect sizes are reported as partial eta squared (η^2^) where applicable.

Permutation testing (5,000 iterations) was used to assess whether Session 3 neurofeedback effects could arise by chance. Session labels were permuted within control dyads while preserving block structure, and the observed contrast coefficient was compared against the resulting null distribution.

Exact n values, statistical tests, degrees of freedom, effect sizes, and p values are reported in the results and corresponding figure legends. Error bars represent standard deviation (SD) unless otherwise specified. Biological units correspond to dyads, and repeated measurements within dyads were treated as non-independent observations.

## Supplemental information

Document S1. Figures S1 and S2, Tables S1–S7, and supplemental analysis

Document S2. CRED-nf checklist summary
