# Supplementary material for: Shaping inter-brain plasticity: A feasibility study of enhancing inter-brain synchrony with dyadic neurofeedback
Source: iScience. 2026 Feb 4;29(3):114894. doi: 10.1016/j.isci.2026.114894 (PMC12927069; doi:10.1016/j.isci.2026.114894)
Supplement: Document S1. Figures S1 and S2, Tables S1–S7, and supplemental analysis [file mmc1.pdf]

iScience, Volume *29*

Supplemental information

Shaping inter-brain plasticity: A feasibility study of enhancing inter-brain synchrony with dyadic neurofeedback

Mario Francis, Andrey Markus, Fine Stuhr-Wulff, and Simone Shamay-Tsoory

Supplementary Analysis

The permutation procedure

To evaluate whether the Session-3 effect reflects chance rather than learningwe contrasted Session 3 against the mean of Sessions 1 and 2 in the NFB group and extracted the coefficient of this contrast. This contrast (weights: –0.5, –0.5, +1) was chosen because it directly captures the observed pattern in the NFB data, where Session 3 consistently exceeded the earlier sessions. The contrast was estimated using a linear mixed-effects model in which the contrast weights served as the predictor and ΔWTC (neurofeedback-related synchrony) was the dependent variable. The model structure matched our primary LME analyses, with Block included as a covariate to control for within-session variance and Dyad entered as a random intercept.

To test whether this effect could occur by chance, we generated a null distribution by permuting session labels (i.e., session order) within Control dyads, while preserving block structure for each session, for 5,000 iterations. For each permutation, the same model was refit and the contrast coefficient extracted, resulting in a null distribution of coefficients. The observed NFB contrast fell in the extreme right tail of this distribution ($p = 0.0314$), providing >95% confidence that the Session 3 effect is not a random occurrence but instead reflects a systematic, emergent pattern specific to the NFB group.

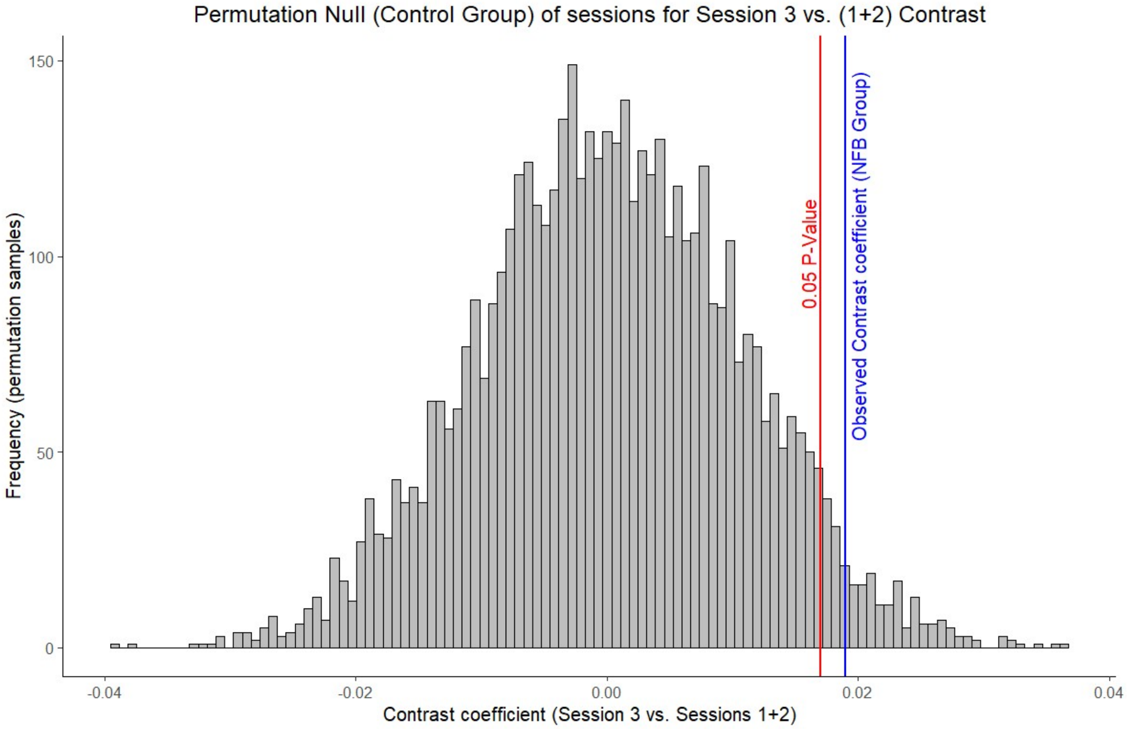

Figure S1. Null distribution of the Session 3 vs. Sessions 1+2 contrast (Control-based permutations). The contrast was estimated using a linear mixed-effects model (contrast weights –0.5, –0.5, +1; ΔWTC as dependent variable; Block as covariate; Dyad as random intercept). Session labels were permuted within Control dyads 5,000 times to generate the null distribution of contrast coefficients. The vertical blue line marks the observed NFB contrast, while the red line marks the p-value of 0.05.

Supplementary tables

ROIcomb	Group	Trend	SE	df	t.ratio	p.value	
lDLPFC_lDLPFC	Control	-0.00079	0.0049	56537	-0.16	0.8725	
	NFB	0.00326	0.00499	56539	0.654	0.5132	
	Contrast	-0.00405	0.00699	56538	-0.579	0.5627	
lDLPFC_mPFC	Control	0.00139	0.00358	56538	0.39	0.6969	
	NFB	0.00696	0.00372	56543	1.872	0.0613	
	Contrast	-0.00557	0.00516	56541	-1.079	0.2806	
lIFG_lDLPFC	Control	-0.00208	0.00346	56537	-0.601	0.5481	
	NFB	0.00736	0.00353	56541	2.087	0.0369	*
	Contrast	-0.00944	0.00494	56539	-1.91	0.0562	
lIFG_lIFG	Control	-0.0228	0.0049	56537	-4.651	0.0001	*
	NFB	-0.00355	0.00499	56539	-0.711	0.477	
	Contrast	-0.01924	0.00699	56538	-2.753	0.0059	*
lIFG_lIPL	Control	-0.009	0.00355	56537	-2.533	0.0113	*
	NFB	3.18e-05	0.00355	56541	0.009	0.9928	
	Contrast	-0.00903	0.00502	56539	-1.797	0.0723	
lIFG_mPFC	Control	-0.0249	0.00358	56538	-6.965	0.0001	*
	NFB	-0.00237	0.00372	56543	-0.638	0.5233	
	Contrast	-0.02253	0.00516	56541	-4.368	0.0001	*
lIFG_rDLPFC	Control	-0.00802	0.00349	56538	-2.3	0.0214	*
	NFB	0.00793	0.00355	56541	2.232	0.0256	*
	Contrast	-0.01595	0.00498	56539	-3.204	0.0014	*
lIFG_rIPL	Control	-0.0111	0.00355	56537	-3.128	0.0018	*
	NFB	0.0133	0.0036	56542	3.684	0.0002	*
	Contrast	-0.02436	0.00506	56540	-4.82	0.0001	*
lIPL_lDLPFC	Control	-0.00493	0.00355	56537	-1.389	0.165	
	NFB	0.00167	0.00355	56541	0.47	0.6384	
	Contrast	-0.0066	0.00502	56539	-1.314	0.1888	
lIPL_lIPL	Control	-0.0257	0.00516	56537	-4.981	0.0001	*
	NFB	0.0121	0.00506	56539	2.384	0.0171	*
	Contrast	-0.03774	0.00722	56538	-5.225	0.0001	*
lIPL_mPFC	Control	0.00465	0.00367	56539	1.265	0.206	

	NFB	0.00274	0.00375	56543	0.73	0.4653	
	Contrast	0.00191	0.00525	56541	0.364	0.7161	
lIPL_rDLPFC	Control	-0.00432	0.00357	56538	-1.208	0.2269	
	NFB	0.0157	0.00355	56541	4.429	0.0001	*
	Contrast	-0.02005	0.00504	56539	-3.979	0.0001	*
mPFC_mPFC	Control	-0.00776	0.00516	56538	-1.502	0.133	
	NFB	0.00125	0.00548	56541	0.228	0.82	
	Contrast	-0.00901	0.00753	56540	-1.196	0.2317	
rDLPFC_lDLPFC	Control	-0.0049	0.00349	56538	-1.405	0.16	
	NFB	0.00486	0.00353	56541	1.379	0.1679	
	Contrast	-0.00976	0.00496	56539	-1.969	0.049	*
rDLPFC_mPFC	Control	0.00263	0.0036	56539	0.729	0.4657	
	NFB	0.00277	0.00372	56543	0.744	0.4566	
	Contrast	-0.00014	0.00518	56541	-0.027	0.9784	
rDLPFC_rDLPFC	Control	-0.011	0.00496	56538	-2.227	0.026	*
	NFB	-0.00025	0.00499	56539	-0.049	0.9608	
	Contrast	-0.0108	0.00703	56538	-1.536	0.1245	
rIFG_lDLPFC	Control	-0.0025	0.00346	56537	-0.723	0.4698	
	NFB	0.00126	0.00353	56541	0.359	0.7198	
	Contrast	-0.00377	0.00494	56539	-0.763	0.4458	
rIFG_lIFG	Control	-0.00146	0.00346	56537	-0.422	0.6733	
	NFB	0.00768	0.00353	56541	2.177	0.0295	*
	Contrast	-0.00914	0.00494	56539	-1.848	0.0646	
rIFG_lIPL	Control	-0.0106	0.00355	56537	-2.997	0.0027	*
	NFB	0.011	0.00355	56541	3.086	0.002	*
	Contrast	-0.0216	0.00502	56539	-4.301	0.0001	*
rIFG_mPFC	Control	-0.00281	0.0036	56538	-0.781	0.4346	
	NFB	0.00139	0.00372	56543	0.374	0.7086	
	Contrast	-0.0042	0.00517	56540	-0.812	0.4168	
rIFG_rDLPFC	Control	-0.00505	0.00349	56538	-1.449	0.1474	
	NFB	0.0104	0.00353	56541	2.958	0.0031	*
	Contrast	-0.01548	0.00496	56539	-3.122	0.0018	*
rIFG_rIFG	Control	0.00521	0.0049	56537	1.064	0.2874	
	NFB	-0.00236	0.00499	56539	-0.474	0.6356	
	Contrast	0.00757	0.00699	56538	1.084	0.2785	
rIFG_rIPL	Control	-0.0132	0.00355	56537	-3.716	0.0002	*
	NFB	0.0131	0.0036	56542	3.642	0.0003	*
	Contrast	-0.0263	0.00506	56540	-5.203	0.0001	*
rIPL_lDLPFC	Control	-0.00616	0.00355	56537	-1.732	0.0832	
	NFB	0.000373	0.00362	56542	0.103	0.9179	
	Contrast	-0.00653	0.00507	56540	-1.287	0.1981	
rIPL_lIPL	Control	-0.0269	0.00365	56537	-7.39	0.0001	*
	NFB	0.00758	0.00363	56543	2.09	0.0366	*
	Contrast	-0.03452	0.00514	56540	-6.713	0.0001	*
rIPL_mPFC	Control	-0.00769	0.00367	56539	-2.095	0.0362	*
	NFB	-0.00462	0.00381	56543	-1.214	0.2248	
	Contrast	-0.00307	0.00529	56541	-0.58	0.5618	
rIPL_rDLPFC	Control	-0.0152	0.00357	56538	-4.241	0.0001	*

	NFB	-0.00277	0.0036	56542	-0.769	0.4417	
	Contrast	-0.01239	0.00507	56540	-2.444	0.0145	*
rIPL_rIPL	Control	-0.0158	0.00516	56537	-3.065	0.0022	*
	NFB	-0.00401	0.0052	56541	-0.771	0.4407	
	Contrast	-0.0118	0.00732	56539	-1.611	0.1072	

Table S1 – This table displays the trends for ROI combinations where at least one group exhibits a significant trend. The trends represent the changes in inter-brain WTC Delta across sessions between groups for each ROI combination.

**ROIcomb**	**Group**	**Trend**	**SE**	**df**	**t.ratio**	**p.value**	
lDLPFC_mPFC	Control	-0.00018	0.00326	97285	-0.056	0.9556	
	NFB	-0.01088	0.00339	97288	-3.211	0.0013	*
	Contrast	0.010702	0.0047	97287	2.276	0.0228	*
lIFG_lDLPFC	Control	0.005548	0.00316	97284	1.757	0.0789	
	NFB	0.009912	0.00321	97287	3.084	0.002	*
	Contrast	-0.00436	0.00451	97285	-0.969	0.3327	
lIFG_lIPL	Control	0.016447	0.00324	97284	5.081	0.0001	*
	NFB	0.017039	0.00324	97287	5.263	0.0001	*
	Contrast	-0.00059	0.00458	97286	-0.129	0.8972	
lIFG_mPFC	Control	0.001183	0.00326	97285	0.363	0.7166	
	NFB	-0.00423	0.00339	97288	-1.248	0.2122	
	Contrast	0.005412	0.0047	97287	1.151	0.2498	
lIFG_rDLPFC	Control	-0.00384	0.00318	97284	-1.207	0.2274	
	NFB	0.013897	0.00321	97287	4.324	0.0001	*
	Contrast	-0.01773	0.00452	97286	-3.924	0.0001	*
lIFG_rIPL	Control	-0.0003	0.00324	97284	-0.093	0.9256	
	NFB	0.019522	0.00328	97287	5.953	0.0001	*
	Contrast	-0.01982	0.00461	97286	-4.303	0.0001	*
lIPL_lDLPFC	Control	0.008104	0.00324	97284	2.504	0.0123	*
	NFB	0.002999	0.00324	97287	0.926	0.3543	
	Contrast	0.005105	0.00458	97286	1.115	0.2648	
lIPL_mPFC	Control	0.006517	0.00335	97285	1.947	0.0515	
	NFB	-0.01003	0.00342	97288	-2.933	0.0034	*
	Contrast	0.016541	0.00478	97287	3.458	0.0005	*
lIPL_rDLPFC	Control	0.008457	0.00324	97284	2.613	0.009	*
	NFB	0.010176	0.00324	97287	3.143	0.0017	*
	Contrast	-0.00172	0.00458	97286	-0.376	0.7072	
rDLPFC_lDLPFC	Control	-0.01646	0.00318	97284	-5.18	0.0001	*
	NFB	0.013042	0.00321	97287	4.058	0.0001	*
	Contrast	-0.0295	0.00452	97286	-6.528	0.0001	*
rDLPFC_mPFC	Control	-0.00465	0.00328	97285	-1.416	0.1569	
	NFB	-0.00371	0.00339	97288	-1.094	0.2739	
	Contrast	-0.00094	0.00472	97287	-0.198	0.8427	

rIFG_lDLPFC	Control	0.003551	0.00316	97284	1.125	0.2608	
	NFB	-0.00266	0.00321	97287	-0.829	0.4073	
	Contrast	0.006213	0.00451	97285	1.379	0.1678	
rIFG_lIFG	Control	-0.00439	0.00316	97284	-1.39	0.1646	
	NFB	0.004844	0.00321	97287	1.507	0.1318	
	Contrast	-0.00923	0.00451	97285	-2.049	0.0405	*
rIFG_lIPL	Control	0.004259	0.00324	97284	1.316	0.1883	
	NFB	0.010704	0.00324	97287	3.307	0.0009	*
	Contrast	-0.00645	0.00458	97286	-1.408	0.1591	
rIFG_mPFC	Control	0.001013	0.00326	97285	0.311	0.7559	
	NFB	-0.0108	0.00339	97288	-3.185	0.0014	*
	Contrast	0.011809	0.0047	97287	2.511	0.012	*
rIFG_rDLPFC	Control	-0.00139	0.00318	97284	-0.438	0.6615	
	NFB	0.004907	0.00321	97287	1.527	0.1268	
	Contrast	-0.0063	0.00452	97286	-1.394	0.1634	
rIFG_rIPL	Control	0.000638	0.00324	97284	0.197	0.8437	
	NFB	-0.00507	0.00328	97287	-1.547	0.1219	
	Contrast	0.005711	0.00461	97286	1.239	0.2152	
rIPL_lDLPFC	Control	0.004878	0.00324	97284	1.507	0.1318	
	NFB	0.004586	0.00328	97287	1.399	0.1619	
	Contrast	0.000292	0.00461	97286	0.063	0.9495	
rIPL_lIPL	Control	-0.00188	0.00324	97284	-0.581	0.561	
	NFB	0.022714	0.00328	97287	6.927	0.0001	*
	Contrast	-0.0246	0.00461	97286	-5.338	0.0001	*
rIPL_mPFC	Control	0.012886	0.00335	97285	3.85	0.0001	*
	NFB	-0.00386	0.00346	97288	-1.115	0.2648	
	Contrast	0.016744	0.00481	97287	3.478	0.0005	*
rIPL_rDLPFC	Control	-0.00536	0.00324	97284	-1.656	0.0977	
	NFB	0.007692	0.00328	97287	2.346	0.019	*
	Contrast	-0.01305	0.00461	97286	-2.833	0.0046	*

Table S2 - This table displays the trends for ROI combinations where at least one group exhibits a significant trend. The trends represent the changes in intra-brain WTC Delta across sessions between groups for each ROI combination.

ROIcomb	ΔWTC.trend	SE	df	t.ratio	p.value	
lDLPFC_lDLPFC	1.028	0.387	27634	2.653	0.008	*
lDLPFC_mPFC	0.643	0.322	27634	1.998	0.0457	*
lIFG_lDLPFC	0.938	0.296	27634	3.17	0.0015	*
lIFG_lIFG	-0.365	0.503	27634	-0.726	0.4679	
lIFG_lIPL	-0.291	0.312	27635	-0.934	0.3503	
lIFG_mPFC	0.462	0.329	27634	1.403	0.1607	
lIFG_rDLPFC	0.581	0.323	27634	1.798	0.0722	
lIFG_rIPL	0.467	0.299	27635	1.563	0.1181	

lIPL_lDLPFC	0.39	0.308	27634	1.267	0.205	
lIPL_lIPL	-0.553	0.448	27634	-1.233	0.2175	
lIPL_mPFC	0.265	0.335	27634	0.792	0.4283	
lIPL_rDLPFC	0.597	0.341	27635	1.75	0.0802	
mPFC_mPFC	0.286	0.528	27634	0.541	0.5885	
rDLPFC_lDLPFC	0.306	0.311	27635	0.982	0.3263	
rDLPFC_mPFC	0.204	0.313	27634	0.653	0.5137	
rDLPFC_rDLPFC	-0.697	0.424	27634	-1.644	0.1003	
rIFG_lDLPFC	2.138	0.292	27635	7.318	0.0001	*
rIFG_lIFG	0.782	0.329	27634	2.378	0.0174	*
rIFG_lIPL	0.439	0.301	27635	1.46	0.1443	
rIFG_mPFC	1.107	0.314	27634	3.523	0.0004	*
rIFG_rDLPFC	0.86	0.291	27634	2.951	0.0032	*
rIFG_rIFG	1.539	0.432	27634	3.563	0.0004	*
rIFG_rIPL	0.913	0.303	27634	3.018	0.0025	*
rIPL_lDLPFC	0.379	0.29	27634	1.307	0.1914	
rIPL_lIPL	-1.239	0.317	27634	-3.905	0.0001	*
rIPL_mPFC	0.5	0.327	27634	1.532	0.1255	
rIPL_rDLPFC	-0.174	0.336	27634	-0.519	0.6041	
rIPL_rIPL	-0.171	0.509	27634	-0.335	0.7373	

Table S3 - This table displays the significant trends for ROI combinations. The trends represent the changes in connectedness predicted by inter-brain WTC Delta for each ROI combination.

ROIcomb	ΔWTC.trend	SE	df	t.ratio	p.value	
lDLPFC_mPFC	0.16286	0.221	47571	0.737	0.4612	
lIFG_lDLPFC	0.6086	0.207	47571	2.939	0.0033	*
lIFG_lIPL	1.37328	0.214	47570	6.415	0.0001	*
lIFG_mPFC	0.64206	0.216	47571	2.967	0.003	*
lIFG_rDLPFC	0.50838	0.201	47571	2.535	0.0112	*
lIFG_rIPL	0.06525	0.227	47569	0.288	0.7734	
lIPL_lDLPFC	0.79746	0.204	47572	3.912	0.0001	*
lIPL_mPFC	-0.24928	0.219	47571	-1.138	0.2552	
lIPL_rDLPFC	-0.00216	0.219	47571	-0.01	0.9922	
rDLPFC_lDLPFC	1.56218	0.212	47570	7.378	0.0001	*
rDLPFC_mPFC	-0.26795	0.228	47570	-1.173	0.2409	
rIFG_lDLPFC	1.38532	0.237	47570	5.84	0.0001	
rIFG_lIFG	0.69932	0.2	47571	3.494	0.0005	*
rIFG_lIPL	0.80479	0.207	47570	3.881	0.0001	*
rIFG_mPFC	0.33009	0.242	47569	1.366	0.1719	
rIFG_rDLPFC	1.44839	0.229	47570	6.335	0.0001	*

rIFG_rIPL	0.56922	0.231	47570	2.466	0.0137	*
rIPL_lDLPFC	-0.19042	0.223	47571	-0.854	0.3933	
rIPL_lIPL	0.18557	0.206	47570	0.902	0.3673	
rIPL_mPFC	-0.21385	0.228	47570	-0.94	0.3473	
rIPL_rDLPFC	-0.69754	0.222	47569	-3.14	0.0017	*

Table S4 - This table displays significant trends for ROI combinations. The trends represent the changes in connectedness predicted by intra-brain WTC Delta for each ROI combination.

Source	Detector	Channel type	Source coordinates	Detector coordinates	ROI_Name
1	1	Long	54.021,69.084,-17.587	50.512,66.33,16.939	rIFG
1	2	Long	54.021,69.084,-17.587	70.103,43.768,-18.472	rIFG
1	5	Long	54.021,69.084,-17.587	29.184,85.158,-13.797	rDLPFC
2	1	Long	64.38,50.37,10.803	50.512,66.33,16.939	rIFG
2	2	Long	64.38,50.37,10.803	70.103,43.768,-18.472	rIFG
2	3	Long	64.38,50.37,10.803	51.167,56.349,37.498	rIFG
2	4	Long	64.38,50.37,10.803	76.997,20.379,19.465	rIFG
2	16	Short	64.38,50.37,10.803	68.72261,43.419022,10.189934	rIFG
3	1	Long	35.272,78.97,16.408	50.512,66.33,16.939	rDLPFC
3	3	Long	35.272,78.97,16.408	51.167,56.349,37.498	rDLPFC
3	5	Long	35.272,78.97,16.408	29.184,85.158,-13.797	rDLPFC
3	8	Long	35.272,78.97,16.408	-0.04,83.39,31.683	mPFC
4	3	Long	62.315,24.986,53.08	51.167,56.349,37.498	rIFG
4	4	Long	62.315,24.986,53.08	76.997,20.379,19.465	rIFG
4	17	Short	62.315,24.986,53.08	65.969467,17.798592,52.831757	rIFG
5	6	Long	69.701,-9.782,63.617	69.044,-46.885,66.427	rIPL
6	6	Long	82.818,-45.959,28.546	69.044,-46.885,66.427	rIPL
6	7	Long	82.818,-45.959,28.546	71.064,-77.654,26.372	rIPL
6	18	Short	82.818,-45.959,28.546	80.920258,-53.529499,27.989861	rIPL
7	6	Long	58.143,-80.514,56.992	69.044,-46.885,66.427	rIPL
7	7	Long	58.143,-80.514,56.992	71.064,-77.654,26.372	rIPL
8	6	Long	38.78,-47.709,93.218	69.044,-46.885,66.427	rIPL
8	19	Short	38.78,-47.709,93.218	36.148842,-55.54166,92.631805	rIPL
9	9	Long	-54.498,68.788,-18.293	-70.55,42.879,-19.263	lIFG
9	12	Long	-54.498,68.788,-18.293	-29.23,85.182,-14.189	lDLPFC
9	13	Long	-54.498,68.788,-18.293	-51.04,65.913,16.492	lIFG
10	9	Long	-64.987,49.59,10.244	-70.55,42.879,-19.263	lIFG
10	10	Long	-64.987,49.59,10.244	-51.83,55.753,37.164	lIFG

10	11	Long	-64.987,49.59,10.244	-77.435,19.001,18.879	lIFG
10	13	Long	-64.987,49.59,10.244	-51.04,65.913,16.492	lIFG
10	20	Short	-64.987,49.59,10.244	-60.44416,56.367661,10.580488	lIFG
11	8	Long	-35.609,78.819,16.092	-0.04,83.39,31.683	mPFC
11	10	Long	-35.609,78.819,16.092	-51.83,55.753,37.164	lDLPFC
11	12	Long	-35.609,78.819,16.092	-29.23,85.182,-14.189	lDLPFC
11	13	Long	-35.609,78.819,16.092	-51.04,65.913,16.492	lDLPFC
12	10	Long	-63.1,23.879,52.682	-51.83,55.753,37.164	lIFG
12	11	Long	-63.1,23.879,52.682	-77.435,19.001,18.879	lIFG
12	21	Short	-63.1,23.879,52.682	-59.80986,30.189224,52.806992	lIFG
13	14	Long	-70.634,-11.463,63.077	-69.286,-48.011,65.668	lIPL
14	14	Long	-39.429,-48.345,92.764	-69.286,-48.011,65.668	lIPL
14	22	Short	-39.429,-48.345,92.764	-42.004814,-40.248489,92.320244	lIPL
15	14	Long	-57.976,-81.286,56.214	-69.286,-48.011,65.668	lIPL
15	15	Long	-57.976,-81.286,56.214	-70.586,-78.617,25.663	lIPL
16	14	Long	-82.542,-47.309,27.8	-69.286,-48.011,65.668	lIPL
16	15	Long	-82.542,-47.309,27.8	-70.586,-78.617,25.663	lIPL
16	23	Short	-82.542,-47.309,27.8	-84.257439,-39.268944,27.089636	lIPL

Table S5 – Optode coordinates that were determined using the Aurora Program by NIRx. To create these optode placements, the fNIRS Optodes' Location Decider (fOLD) [72] was used based on the specified ROIs.

Landmark	Coordinates
Nz	0.4,85.9,-47.6
Iz	0.2,-120.5,-25.8
LPA	-83.8,-18.6,-57.2
RPA	83.9,-16.6,-56.7
Cz	-0.461,-8.416,101.365
Fz	-0.235,62.434,65.513

Table S6 – Landmarks coordinates of 10-10 system that were determined using the Aurora Program by NIRx.

Demographic characteristic	Dyadic neurofeedback (NFB)	Control	Total
**N (participants)**	44	44	88
**Age range (years)**	18–35	18–35	18–35
**Mean age ± SD**	23.93 ± 4.85	23.80 ± 3.60	23.86 ± 4.25
**Female, n (%)**	38 (86.4%)	34 (77.3%)	72 (81.8%)
**Male, n (%)**	6 (13.6%)	10 (22.7%)	16 (18.2%)
**Mother tongue – Hebrew, n (%)**	19 (43.2%)	24 (54.5%)	43 (48.9%)
**Mother tongue – Arabic, n (%)**	25 (56.8%)	20 (45.5%)	45 (51.1%)

Table S7. Demographic characteristics of the analyzed participants. Demographics are reported only for participants included in the final analyses. Race and ethnicity were not formally assessed; participants reported their mother tongue as part of the demographic questionnaire

Supplementary Figures

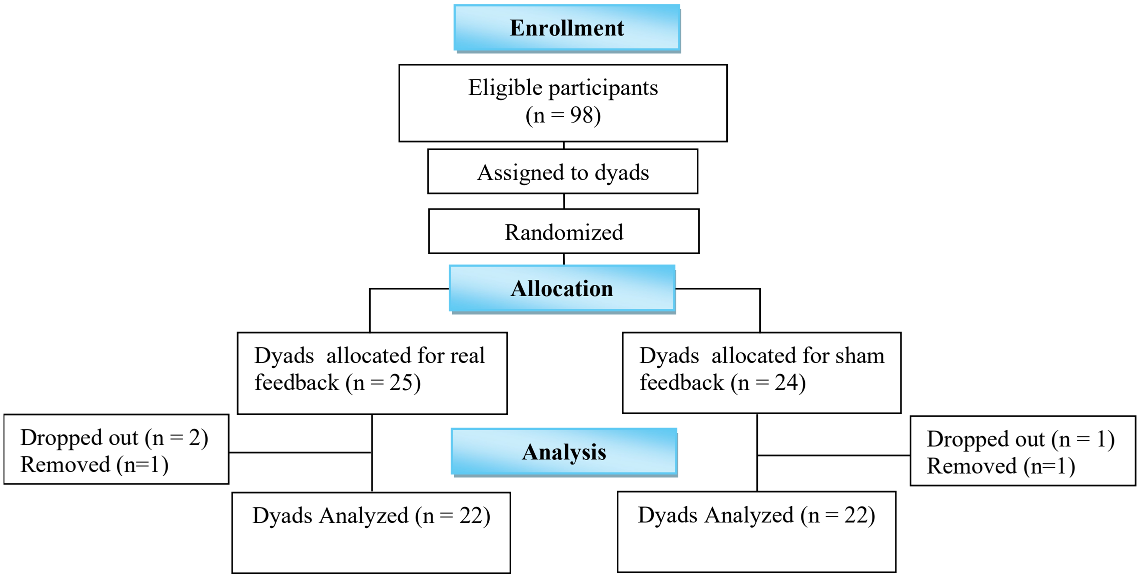

Figure S2 - Consort Diagram: Ninety-eight participants were recruited from the University of Haifa student and Haifa research communities. The participants were randomly assigned upon confirmation of all inclusion criteria to either the dyadic experimental group (25 same-sex dyads; 4 male, 20 female) or the control group (23 same-sex dyads; 3 male, 21 female). The desired sample was 44 dyads (22 each group) based on power analysis, assuming small to medium effect sizes ($\eta^2 = 0.04$), $\alpha = .05$, a power of at least .95, and a correlation of .5 between repeated measures. For inclusion in the study, participants had to be over 18 years old, without any neurological or mental disorder history, and willing and able to provide informed consent. Exclusion criteria included having a dominant left hand, current substance abuse or dependence, significant medical illness or unstable medical conditions that may impact study outcomes, and inability to provide informed consent or comply with study procedures. Three dyads were excluded due to participant dropout by one or both members, and two additional dyads were removed from the analysis because one participant consistently arrived late to every session, creating a tense and potentially confounding atmosphere. As a result, 22 dyads were included in the final analysis for each group.
