# Supplementary material for: Shaping inter-brain plasticity: A feasibility study of enhancing inter-brain synchrony with dyadic neurofeedback
Source: iScience. 2026 Feb 4;29(3):114894. doi: 10.1016/j.isci.2026.114894 (PMC12927069; doi:10.1016/j.isci.2026.114894)
Supplement: Document S2. CRED-nf checklist summary [file mmc2.pdf]

CRED-nf checklist summary

30 June, 2025

Manuscript title: Shaping inter-brain plasticity: enhancing inter-brain synchrony with dyadic neurofeedback
Corresponding Author: Mario Francis
Corresponding author email: mario.f_97@hotmail.com

Item No.	Checklist item	Manuscript Details
**Pre-experiment**		
1a	Pre-register experimental protocol and planned analyses	*This experiment was not preregistered*
1b	Justify sample size	Ninety-eight participants were recruited from the University of Haifa student community. Six participants did not attend one of the sessions and were therefore excluded. The remaining 92 participants (41 with Arabic as their native language, 51 with Hebrew) were randomly assigned—upon confirmation of all inclusion criteria—to either the dyadic experimental group (23 same-sex dyads; 4 male, 19 female) or the control group (23 same-sex dyads; 2 male, 21 female). The desired sample was 44 dyads (22 each group) based on power analysis, assuming small to medium effect sizes (2 = 0.04), =.05, a power of at least .95, and a correlation of .5 between repeated measures.
2a	Employ control group(s) or control condition(s)	The control group received sham feedback, where the velocity of the fish was based on time-matched coherence values from a different dyad. This approach ensured the feedback appeared realistic while remaining unrelated to the participants' actual brain activity.
2b	When leveraging experimental designs where a double-blind is possible, use a double-blind	*The experiment did not include a double-blind*
2c	Blind those who rate the outcomes	Feedback was based either on real-time inter-brain coherence (experimental group) or on time-matched prerecorded coherence from another dyad (control group), with participants blinded to their group assignment.
	Blind those who analyse the data	*Those who analysed the data were not blind to group assignment*
2d	Examine to what extent participants and experimenters remain blinded	*No measures were taken to examine whether participants and experimenters remained blind*
2e	In clinical efficacy studies, employ a standard-of-care intervention group as a benchmark for improvement	*NA: This is not a clinical efficacy study*

**Control measures**		
3a	Collect data on psychosocial factors	*Psychosocial factors were not measured*
3b	Report whether participants were provided with a strategy	No specific instructions regarding mental strategy were given, because neurofeedback studies showed that an explicit strategy is not required and may even impede learning to regulate brain activation.
3c	Report the strategies participants used	*The strategies participants used were not recorded or not reported in the manuscript*
3d	Report methods used for online-data processing and artifact correction	Online preprocessing: fNIRS data collected by each of the two NIRx devices during the neurofeedback sessions were streamed for preprocessing to the Turbo-Satori platform, with the pre-processed signal becoming available in near real-time. Pre-processing included online frequency filtering using a bandpass filter with a 0.2 Hz. low cutoff and a 0.01 Hz. high cutoff frequencies, and short-separation regression on all available fNIRS channels. Preprocessing aimed at removing extracerebral physiological signal artifacts, such as those resulting from heartbeat and respiration. Additionally, Turbo-Satori converted raw light intensity values collected by the NIRx devices into relative O Hb and HHb concentrations using the modified Beer–Lambert law 78,79. Subsequent to this, a preprocessed data stream was generated, which was used in neurofeedback signal computation. This online preprocessing was applied only to the IFG signal, the target of neurofeedback, as extending it to all measured regions would delay the feedback, which is undesirable for the learning process. Therefore, offline preprocessing was performed later for the other regions. Offline Data Preprocessing: Offline data pre-processing was done using the Satori by Brain innovation. The process began with the conversion of raw intensity data into optical d
		ensity values. Motion artifacts were then corrected using TDDR and Spike removal. A bandpass filter (0.01–0.2 Hz) was applied to remove physiological noise, such as heart rate and respiration, while retaining the hemodynamic signals. Optical density data were subsequently converted into O Hb and HHb concentrations using the modified Beer–Lambert law to extract hemodynamic signals. Channels exhibiting anomalous signals, such as a high positive correlation between O Hb and HHb, were excluded based on criteria established in previous studies. For each channel, short separation regression was carried out using its nearest short separation counterpart.
3e	Report condition and group effects for artifacts	*Condition and group effects for artifacts were not measured, or not reported in the manuscript*

4a	Report how the online-feature extraction was defined	Online preprocessing fNIRS data collected by each of the two NIRx devices during the neurofeedback sessions were streamed for preprocessing to the Turbo-Satori platform, with the pre-processed signal becoming available in near real-time. Pre-processing included online frequency filtering using a bandpass filter with a 0.2 Hz. low cutoff and a 0.01 Hz. high cutoff frequencies, and short-separation regression on all available fNIRS channels. Preprocessing aimed at removing extracerebral physiological signal artifacts, such as those resulting from heartbeat and respiration. Additionally, Turbo-Satori converted raw light intensity values collected by the NIRx devices into relative O Hb and HHb concentrations using the modified Beer–Lambert law 79,80. Subsequent to this, a preprocessed data stream was generated, which was used in neurofeedback signal computation. This online preprocessing was applied only to the IFG signal, the target of neurofeedback, as extending it to all measured regions would delay the feedback, which is undesirable for the learning process. Therefore, offline preprocessing was performed later for the other regions. Coherence computation Custom Matlab (Matlab v.2023b, by Mathworks Ltd.) code was used to combine the preprocessed data streams from both participants in order to calculate coheren
		ce values. The code was designed to run in near-real time. First the code read new timestamped data samples from each data stream as new data frames arrived. Then, only data for the oxygenated hemoglobin (O2Hb) concentrations from the relevant regions of interest (ROIs) was retained from each frame. The ROIs chosen for neurofeedback computation included the left and right Inferior Frontal Gyri (IFG). Data samples related to the IFG of each participant were averaged within each new frame. Samples from the two streams were combined by timestamp into a joint structure, which represented a synchronized dyadic dataset of the IFG of each participant. Coherence values were calculated from this set using Wavelet Transform Coherence (WTC) with a Morlet seed wavelet 71 with wavelengths of between 6 and 30 seconds. The wavelength for WTC analyses were determined from analysis of the control group in a pilot study and were constrained by the length of the computation window. The WTC toolbox 81 for MATLAB was used for this calculation. The calculation was performed at 1 seconds. intervals asynchronously to the arriving data, and a backward window of 30 seconds from the most current dataframe's timestamp was used for each calculation cycle. Since data frames arrived at a frequency of ~5 samples-per-second, each new calcula

		tion cycle included between 4-6 new data samples, omitting the same number of the oldest samples. Coherence values resulting from each computation cycle were averaged across time and wavelength to produce a single neurofeedback value, which was transmitted to the feedback visualizer for the neurofeedback group. Additionally, the code had the ability to load a data file pertaining to a previous session of a different dyad and transmit coherence values from that file to the feedback visualizer for the control group. Same as the preprocessing the online WTC calculations was applied only to the IFG signal, the target of neurofeedback, as extending it to all measured regions would delay the feedback, which is undesirable for the learning process.
4b	Report and justify the reinforcement schedule	The visual portion of the feedback loop was comprised of a short clip of a fish facing leftwards and moving slightly around the center of the screen, placed against a moving background of sand, rocks and marine flora. This generated an illusion of a fish swimming in the ocean from the right to the left of the display. The speed at which the background moved across was determined by the received neurofeedback values as described in the previous section. Thus, the overall effect was that of a fish swimming faster when coherence values were higher (either real or sham, depending on the group). Both participants in a dyad were seated in front of a single computer monitor presenting this visualization, thus receiving identical concurrent feedback.
4c	Report the feedback modality and content	Reported in section 4.b.
4d	Collect and report all brain activity variable(s) and/or contrasts used for feedback, as displayed to experimental participants	To examine changes in inter-brain synchrony between groups in the neurofeedback-trained region (the mean of left and right IFG), a mixed-effects model was employed. We subtracted the mean WTC taken in the pre-training baseline of each session from the WTC means for each training block in that session (ΔWTC), in the left and right IFG ROIs. We analyzed changes in ΔWTC across the three sessions for each group. Block number was included as a fixed factor not included in the interactions, to control for variance, and random intercepts were incorporated for each dyad to account for repeated measures.
4e	Report the hardware and software used	*This field has been left blank*
**Outcome measures - brain**		

5a	Report neurofeedback regulation success based on the feedback signal	To examine changes in inter-brain synchrony between groups in the neurofeedback-trained region (the mean of left and right IFG), a mixed-effects model was employed. We subtracted the mean wavelet transform coherence (WTC) taken in the pre-training baseline of each session from the WTC means for each training block in that session (ΔWTC), in the left and right IFG ROIs. We analyzed changes in ΔWTC across the three sessions for each group. Block number was included as a fixed factor not included in the interactions, to control for variance, and random intercepts were incorporated for each dyad to account for repeated measures. A Type II Wald ^{2} comparison test between the model with interaction terms and the model without interactions revealed that the inclusion of interactions significantly improved the model's predictive power [$^2(1) = 5.52$, p = 0.019]. Thus, the model with interactions was selected for further analyses. The analysis revealed a significant interaction between group and session (F(1, 1195.37) = 5.51, p = .019, ^{2} = 0.0046), indicating different trends in WTC across sessions for the experimental and control groups (Figure 2a). Follow-up analyses examined trends within each group. For the experimental group, the ΔWTC significantly increased across sessions, with a positive trend (M = 0.01026, SE
		= 0.0037, t(1196) = 2.77, p = .006). In contrast, for the control group, no significant change in ΔWTC was observed (M = -0.0019, SE = 0.00359, t(1194) = -0.52, p = .61). To further investigate the source of the significant trend, the session variable was analyzed as a categorical factor using the same linear mixed-effects model. The results revealed a significant interaction between Group and Session [F(2, 1192) = 5.57, p = 0.004, ^{2} = 0.01], thus post hoc analyses were conducted (Figure 2b). In Session 1, there was no significant difference (M = -0.015, SE = 0.0140, t(63.6) = -1.089, p = 0.2804) between the Control group (M = -0.0084, SD = 0.0098) and the NFB group (M = 0.007, SD = 0.01). Similarly, in Session 2, the Control group (M = -0.015, SD = 0.0098) and the NFB group (M = -0.011, SD = 0.0098) showed no significant difference (M = -0.00489, SE = 0.0139, t(61.9) = -0.352, p = 0.7257). In contrast, Session 3 revealed a significant difference between the Control group (M = -0.012, SD = 0.0100) and the NFB group (M = -0.03787, SE = 0.0139, t(61.4) = -2.734, p = 0.0082) with the NFB group demonstrating a notably higher mean of ΔWTC. To verify that there were no baseline differences in inter-brain synchrony between groups prior to neurofeedback training, a one-way ANOVA was conducted on the first baseline
		measurement. The analysis revealed no significant difference in inter-brain synchrony between the groups at baseline (F(1, 418) = 1.12, p = .29).
5b	Plot within-session and between-session regulation blocks of feedback variable(s), as well as pre-to-post resting baselines or contrasts	Reported in section 5.a.

5c	Statistically compare the experimental condition/group to the control condition(s)/group(s) (not only each group to baseline measures)	Reported in section 5.a.
**Outcome measures - behaviour**		
6a	Include measures of clinical or behavioural significance, defined a priori, and describe whether they were reached	To examine changes in social connectedness over time, we conducted a mixed-effects model analysis with fixed effects of group (experimental vs. control) and measurement (four measurements in total: pre-training and post-training in Sessions 1, 2, and 3); random intercepts were included for participants nested within dyads to account for repeated measures; the predicted factor was each participant's mean score of the items in the connectedness questionnaire (connectedness mean) taken at each measurement. A Type II Wald ^{2} comparison test between the model with interaction terms and the model without interactions revealed that the inclusion of interactions significantly improved the model's predictive power [2(1) = 4.9; p = 0.027]. Thus, the model which included interactions was selected for further analyses. Overall, the connectedness mean showed a significant increase in social connectedness across time points (F(1, 258.38) = 26.51, p = .001, ^{2} = 0.09). This general increase was observed in both groups (Figure 1a), but the interaction between group and measurement was significant (F(1, 258.38) = 4.90, p = .028, ^{2} = 0.02). Follow-up analyses revealed that while both groups exhibited positive trends, the experimental group demonstrated greater improvement (M = 0.460, SE = 0.089, t(258) = 5.18, p = .001) comp
		ared to the control group (M = 0.183, SE = 0.088, t(258) = 2.09, p = .038). Further analysis of individual items revealed different patterns in participants' responses in each item (Figure 1b). When asked to what extent they felt connected to the other person or for their willingness to participate in another experiment with the same partner, the interaction was not significant for either question (F(1, 258.84) = 0.36, p = .55, ^{2} = 0.001) and (F(1, 170.50) = 3.20, p = .075, ^{2} = 0.02) respectively. For the question assessing participants' desire to get to know the other person better, a significant group by measurement interaction was detected (F(1, 258.20) = 5.49, p = .020, ^{2} = 0.02). This interaction reflected a marked positive trend in the experimental group (M = 0.394, SE = 0.097, t(258) = 4.06, p = .001), whereas the control group showed no significant change (M = 0.074, SE = 0.096, t(258) = 0.78, p = .44) over time. Similarly, for the question assessing the extent to which participants felt they could understand the other person, a significant group by measurement interaction was observed (F(1, 258.39) = 5.85, p = .016, ^{2} = 0.02). This interaction was driven by a significant increase in the experimental group (M = 0.338, SE = 0.111, t(259) = 3.05, p = .003), while no change was evident in the control

		group (M = -0.039, SE = 0.110, t(258) = -0.36, p = .72). For feelings of identifying with the other person, the group by measurement interaction also reached significance (F(1, 258) = 4.69, p = .031, ^{2} = 0.02). This interaction reflected a marginally positive trend in the experimental group (M = 0.180, SE = 0.093, t(258) = 1.95, p = .052), compared to no significant change in the control group (M = -0.101, SE = 0.091, t(258) = -1.11, p = .27). Finally, for the question assessing the extent to which the activity strengthened participants' connection with the other person, the group by measurement interaction was significant (F(1, 169.33) = 7.49, p = .007, ^{2} = 0.04). This interaction reflected a significant decrease over time in the control group (M = -0.671, SE = 0.186, t(170) = -3.60, p = .001), while no significant trend was observed in the experimental group (M = 0.057, SE = 0.189, t(171) = 0.30, p = .77). To ensure there were no baseline differences in perceived connectedness between groups, a one-way ANOVA was conducted comparing the Neurofeedback and Control groups at the first measurement. The analysis revealed no significant difference in baseline connectedness between the groups (F(1, 86) = 0.22, p = .64)
6b	Run correlational analyses between regulation success and behavioural outcomes	The relationship between behavioral change and inter/intra-brain synchrony was examined only in the experimental group, as our primary interest lies in the changes in inter/intra-brain synchrony following the neurofeedback and their association with behavior. To assess the relationship between behavioral change and inter/intra-brain synchrony we created a new variable that represents the change in connectedness. As connectedness was measured 4 times, we created a new variable by subtracting the mean connectedness score for measurements in the preceding measurement from that of measurements 2-4, resulting in three delta connectedness values (Δconn) representing the change in connectedness between each two consecutive sessions (in other words we had 3 values of Δconn: (1) connectedness measurement 2 minus 1 (2) 3 minus 2 (3) 4 minus 3). For inter-brain analysis, LME model was constructed to investigate whether changes in the delta of WTC predicted changes in social connectedness following training. The model included Δconn as the predicted variable; ΔWTC, session (corresponding to Δconn measurement), and ROI combination, as fixed factors; the random effects accounted for the intercepts of dyads. While the primary focus was on the prediction of ΔWTC by ROI combinations regardless of session, session was included

		as a fixed effect to account for variance due to the experimental structure but was excluded from the interactions. A Type II Wald ^{2} comparison test between the model with interaction terms and the model without interactions revealed that the inclusion of interactions significantly improved the model's predictive power [$^2(82) = 318.8$, $p = 0.001$]. Thus, the model with interactions was selected for further analyses. A significant interaction between ROI combination and ΔWTC was found ($F(27, 27634) = 3.4$, $p = .001$, $^2 = 0.00332$), indicating that the combined effects of WTC changes, and ROI combination significantly predicted changes in social connectedness. Post hoc analysis identified specific ROI combinations showing significant trends – (see table S3 and figure 5 panel (a) for the significant ROI combinations).
7a	Upload all materials, analysis scripts, code, and raw data used for analyses, as well as final values, to an open access data repository, when feasible	Data and code availability • Raw and preprocessed data have been deposited at Mendely Data and are publicly available as of the date of publication at https://data.mendeley.com/preview/wyc43xpcff?a=b4922335-e89a-4cfc-a7ea-66ceb1069544 • All original code has been deposited Mendely Data and are publicly available as of the date of publication at https://data.mendeley.com/preview/wyc43xpcff?a=b4922335-e89a-4cfc-a7ea-66ceb1069544 • Any additional information required to reanalyze the data reported in this article is available from the lead contact upon request
